# H4K12 Lactylation Regulates NDUFS7 to Drive Microglia Reverse Electron Transport in Spinal Cord Injury

**DOI:** 10.1002/advs.78057

**Published:** 2026-09-29

**Authors:** Chenglong Hong, Zhichen Jiang, Zhouwei Wu, Chenyu Wu, Jiang Liu, Kaijie Guo, Yihui Liang, Shaobo Xu, Yuchen Jin, Yong Xiao, Lei Guo, Hui Xu, Sunren Sheng, Chenggui Wang

**Affiliations:** ^1^ Department of Orthopaedics The Second Affiliated Hospital and Yuying Children's Hospital of Wenzhou Medical University Wenzhou China; ^2^ Zhejiang Provincial Key Laboratory of Orthopaedics Wenzhou China; ^3^ The Second Clinical Medical College of Wenzhou Medical University Wenzhou China; ^4^ Department of Critical Care Medicine The Second Affiliated Hospital and Yuying Children's Hospital of Wenzhou Medical University Wenzhou China

**Keywords:** histone lactylation, LDHA, NDUFS7, PROTAC, reverse electron transport, spinal cord injury

## Abstract

Microglial polarization toward the pro‐inflammatory state drives secondary injury following spinal cord injury (SCI), yet the mechanisms of metabolic reprogramming governing this phenotypic shift remain elusive. Here, we identify a lactate‐dependent signaling axis linking histone lactylation to mitochondrial reverse electron transport (RET) that sustains neuroinflammation. We demonstrate that SCI‐induced accumulation of lactate promotes histone H4 lysine 12 lactylation (H4K12la), which directly upregulates NDUFS7, a core subunit of mitochondrial Complex I. Elevated NDUFS7 triggers mitochondrial hyperactivity and RET, resulting in a reactive oxygen species (ROS) burst that enforces pro‐inflammatory polarization. To intervene in this cascade, we engineered a biomimetic nanotherapeutic, MM@mPTC, comprising an LDHA‐targeting PROTAC encapsulated within ROS‐responsive micelles and coated with microglial membranes (MM). The biomimetic MM@mPTC system actively targets activated microglia and undergoes ROS‐responsive payload release to specifically degrade LDHA. This targeted degradation dismantles the pathogenic “LDHA‐H4K12la‐NDUFS7‐RET” axis, halting RET‐driven ROS production and reprogramming microglia toward a reparative phenotype. Consequently, this intervention significantly mitigates neuroinflammation, preserves neuronal tissue, and promotes robust locomotor recovery, presenting a precise metabolic‐epigenetic therapeutic paradigm for central nervous system trauma.

## Graphical abstract

Lactate accumulation after SCI promotes H4K12la lactylation, which upregulates NDUFS7, triggering mitochondrial RET and ROS, enforcing pro‐inflammatory microglial polarization. MM@mPTC, a biomimetic nanotherapeutic, degrades LDHA in activated microglia, reducing H4K12la lactylation and blocking RET, thus curbing ROS, fostering a reparative phenotype, mitigating neuroinflammation, preserving tissue, and restoring locomotor function.

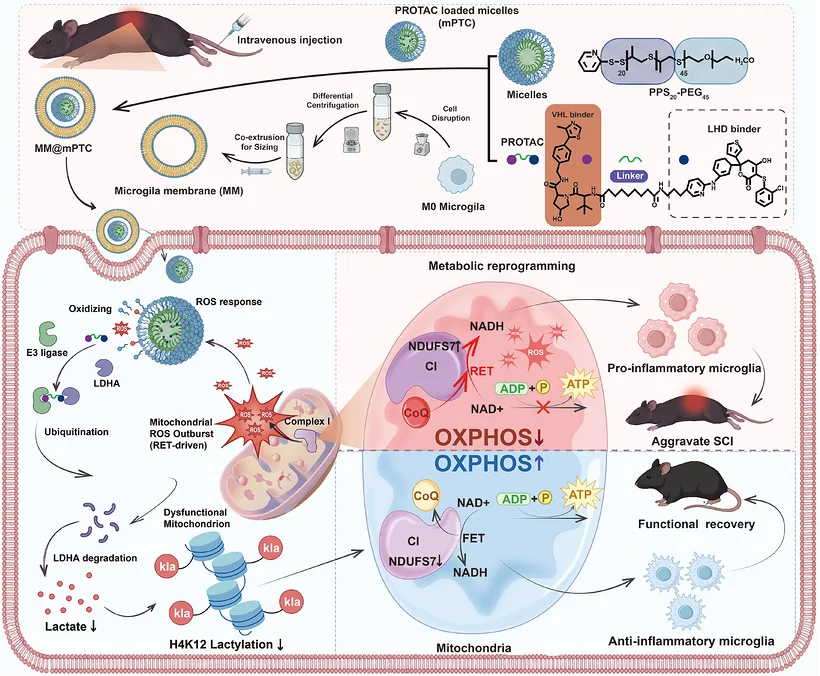

## 1 Introduction

Spinal cord injury (SCI) triggers a detrimental neuroinflammatory response predominantly driven by microglia [1]. The polarization of these cells toward a pro‐inflammatory state is a critical step in amplifying tissue damage [2], yet the underlying drivers remain incompletely understood. Metabolic reprogramming toward glycolysis is a hallmark of inflammation, leading to significant lactate accumulation [3]. Beyond its traditional role as a metabolic by‐product, lactate has recently been identified as a precursor for histone lactylation, a novel epigenetic modification that directly couples cellular metabolic states to gene regulation [4]. While histone lactylation describes a mechanism by which metabolic shifts‐such as the Warburg effect‐are translated into transcriptional outputs [5, 6, 7], its presence, genomic targets, and functional consequences in microglia within the neuroinflammatory environment of the injured spinal cord remain largely uncharted territory.

A key downstream consequence of inflammatory metabolism is mitochondrial dysfunction, particularly the generation of mitochondrial reactive oxygen species (mtROS) via reverse electron transport (RET) at Complex I [8, 9, 10, 11]. RET is a process in the mitochondrial electron transport chain (ETC) where electrons flow in reverse of the normal, energy‐producing direction [12]. Under physiological conditions, forward electron transport (FET) proceeds from higher‐energy donors (like NADH) to lower‐energy acceptors (ultimately oxygen), a process that releases energy used to pump protons and build the proton motive force (Δp) [13]. For FET to occur, the difference in reduction potential between the NAD^+^/NADH and the CoQ/CoQH_2_ couples (ΔEh) must be sufficient for two electrons to pump four protons against Δp such that the thermodynamic requirement for FET is 2ΔEh > 4Δp [14]. Under pathological conditions characterized by dramatic metabolic shifts—most notably during ischemia‐reperfusion injury (IRI) and pro‐inflammatory immune activation—the direction of mitochondrial electron flow can be reversed [8, 15]. Specifically, electrons are driven backward from the reduced CoQ pool to the FMN (flavin mononucleotide) site of Complex I [16]. Here, instead of reducing NAD^+^, electrons leak onto molecular oxygen to generate a burst of superoxide. This reversal occurs when the proton motive force thermodynamically overpowers the redox potential difference (4Δp > 2ΔEh) [17]. Although RET‐driven mtROS fundamentally fuels inflammatory activation [10, 18], the exact mechanisms triggering this process and its functional impact on microglia during SCI remain elusive.

NDUFS7 (NADH dehydrogenase ubiquinone Fe‐S protein 7), a critical core subunit forming the ubiquinone‐binding pocket (Q‐site), is essential for coupling electron transfer to proton translocation [16, 19]. Emerging evidence highlights that subunits forming this Q‐site serve as critical molecular checkpoints; for instance, NDUFS2, which co‐constitutes this binding site, has been identified as the essential redox sensor required to drive mtROS signaling that sustains tumorigenesis and hypoxic adaptation [20, 21]. Drawing from this precedent, we postulate that the overexpression of NDUFS7 may similarly hijack mitochondrial bioenergetics: by enhancing the proton‐pumping capacity of Complex I to lead to an excessive elevation of the mitochondrial membrane potential. This hyperpolarized state creates a thermodynamic threshold that highly favors RET [22]. Thus, it remains to be elucidated whether NDUFS7 acts as a direct molecular trigger that drives RET.

Guided by these mechanistic insights, we engineered a multifunctional biomimetic nanoplatform, MM@mPTC. The core of this system features an LDHA‐targeting Proteolysis‐Targeting Chimera (PROTAC) designed to suppress lactate production at its source [23, 24, 25]. To achieve precise delivery, we synthesized an amphiphilic PEG_45_‐PPS_20_ copolymer to encapsulate the PROTAC. The key innovation lies in the hydrophobic poly (propylene sulfide) (PPS) core, which serves as a highly sensitive ROS switch [26]. Upon exposure to the elevated ROS characteristic of the lesion microenvironment, the PPS block undergoes a rapid hydrophobic‐to‐hydrophilic phase transition via oxidation, triggering micelle disassembly and on‐demand payload release [27]. Furthermore, these micelles are cloaked in a microglial membrane, endowing the system with natural tropism to actively target activated microglia and cross biological barriers [28, 29, 30].

Through this approach, we demonstrate that leveraging the pathological microenvironment to trigger cargo release allows for microglia‐specific anti‐inflammatory therapy. The successful repolarization of microglia via MM@mPTC confirms the critical role of the “LDHA‐H4K12la‐NDUFS7‐RET” cascade in sustaining neuroinflammation. Following treatment in SCI, we observed robust anti‐inflammatory effects and functional recovery, underscoring the translational promise of this system. This work not only elucidates a previously unrecognized epigenetic mechanism driving secondary injury but also highlights the utility of combining PROTAC technology with biomimetic delivery to achieve site‐specific immunomodulation.

## 2 Results

### 2.1 Histone Lactylation and Pro‐Inflammatory Microglia Polarization Were Enhanced after Spinal Cord Injury

To systematically investigate the phenotypic and metabolic landscape in microglia following SCI, we first analyzed public single‐cell RNA sequencing (scRNA‐seq) data from spinal cord tissues. Gene Set Enrichment Analysis (GSEA) confirmed a significant trend of glycolysis enrichment in the overall tissue metabolism post‐SCI (Figure 1A). Comparative analysis further revealed that microglia exhibited the most pronounced glycolytic changes among all cell types in the injured microenvironment (Figure 1B). Subsequent clustering analysis demonstrated that these microglia segregated into distinct resting state, pro‐inflammatory states, and anti‐inflammation states (Figure S1A,B). Consistent with this, we observed a concurrent upregulation of both pro‐inflammatory (CD86, iNOS) and anti‐inflammatory (CD206, Arg‐1) markers in the injured tissue, indicating a coordinated polarization of microglia from a resting state toward distinct pro‐inflammatory and anti‐inflammatory populations after SCI (Figure S1C).

**FIGURE 1:**
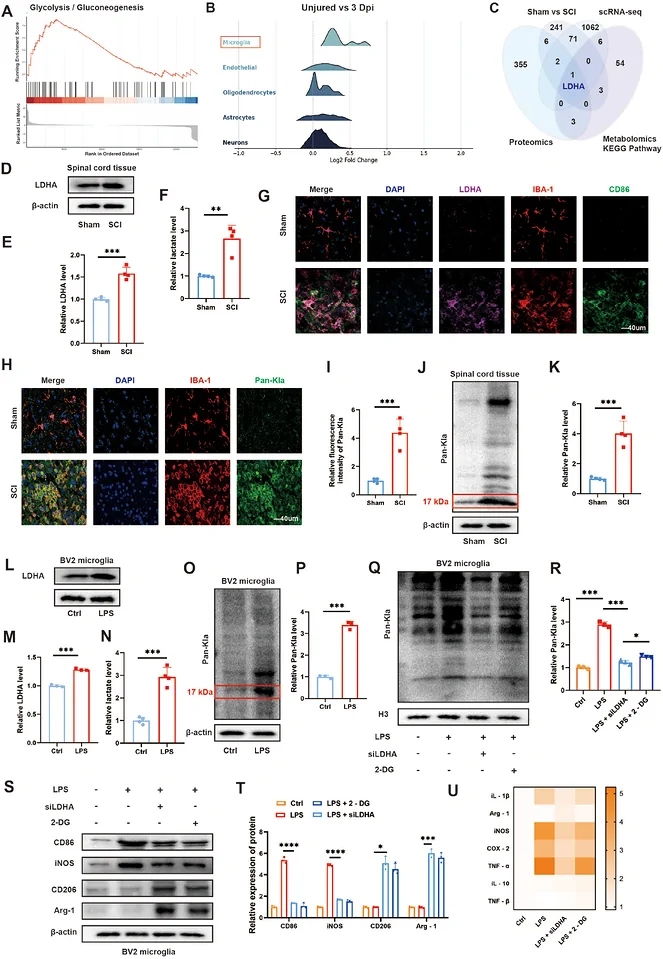
LDHA‐mediated lactate accumulation drives microglial pro‐inflammatory polarization via histone lactylation. (A) Gene Set Enrichment Analysis (GSEA) of the Glycolysis/Gluconeogenesis pathway following SCI. (B) Ridge plot showing the Log2 Fold Change of glycolysis‐related genes across different cell types. (C) Venn diagram showing the intersection of proteomics, transcriptomics (Sham vs SCI, Resting state vs pro‐inflammatory state), and metabolomics/KEGG pathway data. (D‐E) Representative Western blot bands and quantification of LDHA expression in spinal cord tissues from Sham and SCI groups (*n* = 4). (F) Quantitative analysis of relative lactate level in spinal cord tissues (*n* = 4). (G) Representative immunofluorescence images of LDHA (purple), DAPI (blue), CD86 (green), and IBA‐1 (red) in spinal cord tissues (*n* = 4). (H) Representative immunofluorescence images of Pan‐Kla (green) and IBA‐1 (red) in spinal cord tissues from Sham and SCI groups (*n* = 4). (I) Quantification of the relative Pan‐Kla fluorescence intensity in microglia (n = 4). (J‐K) Western blot analysis and quantification of Pan‐Kla levels at the 17 kDa band (*n* = 4). (L‐M) Representative Western blot bands and quantification of LDHA expression in control (Ctrl) and LPS‐stimulated BV2 microglia (*n* = 3). (N) Quantitative analysis of relative lactate level in BV2 microglia (*n* = 4). (O‐P) Western blot analysis and quantification of Pan‐Kla at the 17 kDa band in BV2 microglia (*n* = 3). (Q‐R) Western blot bands and quantification of Pan‐Kla under siLDHA or 2‐DG treatments (*n* = 3). (S‐T) Representative Western blot bands and quantitative analysis of pro‐inflammatory markers (CD86, iNOS) and anti‐inflammatory markers (CD206, Arg‐1) under different treatments (*n* = 3). (U) Heatmap of the relative mRNA expression of polarization markers and inflammatory cytokines across different treatment groups (*n* = 3). Data are presented as mean ± SD. Comparisons were performed using an unpaired *t*‐test (two groups), one‐way ANOVA or two‐way ANOVA with Tukey's post hoc test as appropriate. ^*^ *p* < 0.05, ^**^ *p* < 0.01, ^***^ *p* < 0.001, and ^****^ *p* < 0.0001 vs. indicated group.

Given that microglial polarization is deeply intertwined with metabolic reprogramming, we integrated multi‐dimensional omics data to explore the underlying drivers. By intersecting differentially expressed targets from in vivo tissue transcriptomes (Sham vs. SCI, Figure S1D) with microglial scRNA‐seq (resting microglia vs. pro‐inflammatory microglia, Figure S1E) and in vitro proteomic datasets (resting microglia vs pro‐inflammation microglia, Figure S1F), and cross‐referencing these targets with metabolism‐related KEGG pathways, we precisely pinpointed the key glycolytic enzyme Lactate Dehydrogenase A (LDHA) (Figures 1C and S1D–F). Subsequent KEGG and Gene Ontology (GO) enrichment analyses further confirmed that these pathways are predominantly associated with glycolysis and related metabolic processes (Figure S1G,H). Together, these findings identify LDHA as the core metabolic switch driving the pro‐inflammatory polarization of microglia.

We next validated these findings in a mouse model of SCI. LDHA protein levels were significantly elevated in the injured spinal cord (Figure 1D,E). Consistent with LDHA upregulation, lactate levels in the SCI‐injured tissue were significantly elevated (Figure 1F). Specifically, LDHA intensity was robustly enriched in spinal microglia (IBA‐1^+^ and CD86^+^), indicating a prominent co‐localization between LDHA and CD86 (Figures 1G and S2A,B).

Given the established link between lactate and histone lactylation [31], we hypothesized that elevated lactate might drive lactylation in microglia. Indeed, Pan‐Kla strongly co‐localized with microglia post‐SCI (Figure 1H,I). Furthermore, global lysine lactylation was markedly elevated in the injured spinal cord, characterized by a prominent 17 kDa band enrichment consistent with core histones (Figure 1J,K).

To establish a direct causal link in vitro, we employed BV2 microglia stimulated with LPS to mimic the pro‐inflammatory environment of SCI [32]. LPS treatment significantly increased LDHA expression at both protein and mRNA levels (Figures 1L,M and S2C) and intracellular lactate levels (Figure 1N). This was accompanied by a specific increase in protein lactylation, again with a prominent band at 17 kDa (Figure 1O,P). Crucially, when lactate production was inhibited using either siLDHA or the glycolytic inhibitor 2‐Deoxy‐D‐glucose (2‐DG), the LPS‐induced accumulation of the 17 kDa lactylation signal was markedly attenuated (Figure 1Q,R). Furthermore, the pro‐inflammatory phenotype in LPS‐stimulated microglia was significantly suppressed by targeting lactate metabolism with either siLDHA or 2‐DG (Figure 1S–U). Collectively, these data demonstrate that SCI induces a metabolic shift in microglia toward lactate production, which in turn drives specific protein lactylation‐potentially on histones [3, 33] and actively contributes to a pro‐inflammatory microglial polarization.

### 2.2 H4K12 Lactylation Mediates the Upregulation of NDUFS7 in Pro‐Inflammatory Microglia

To identify the specific histone lactylation involved in microglial activation, we screened a panel of eight common histone lactylation sites in BV2 microglia. Western blot analysis revealed that among the sites tested (H2B16, H3K9, H3K14, H3K18, H4K5, H4K8, H4K12, H4K16), H4K12 lactylation (H4K12la) exhibited the most pronounced increase upon LPS stimulation (Figure 2A,B).

**FIGURE 2:**
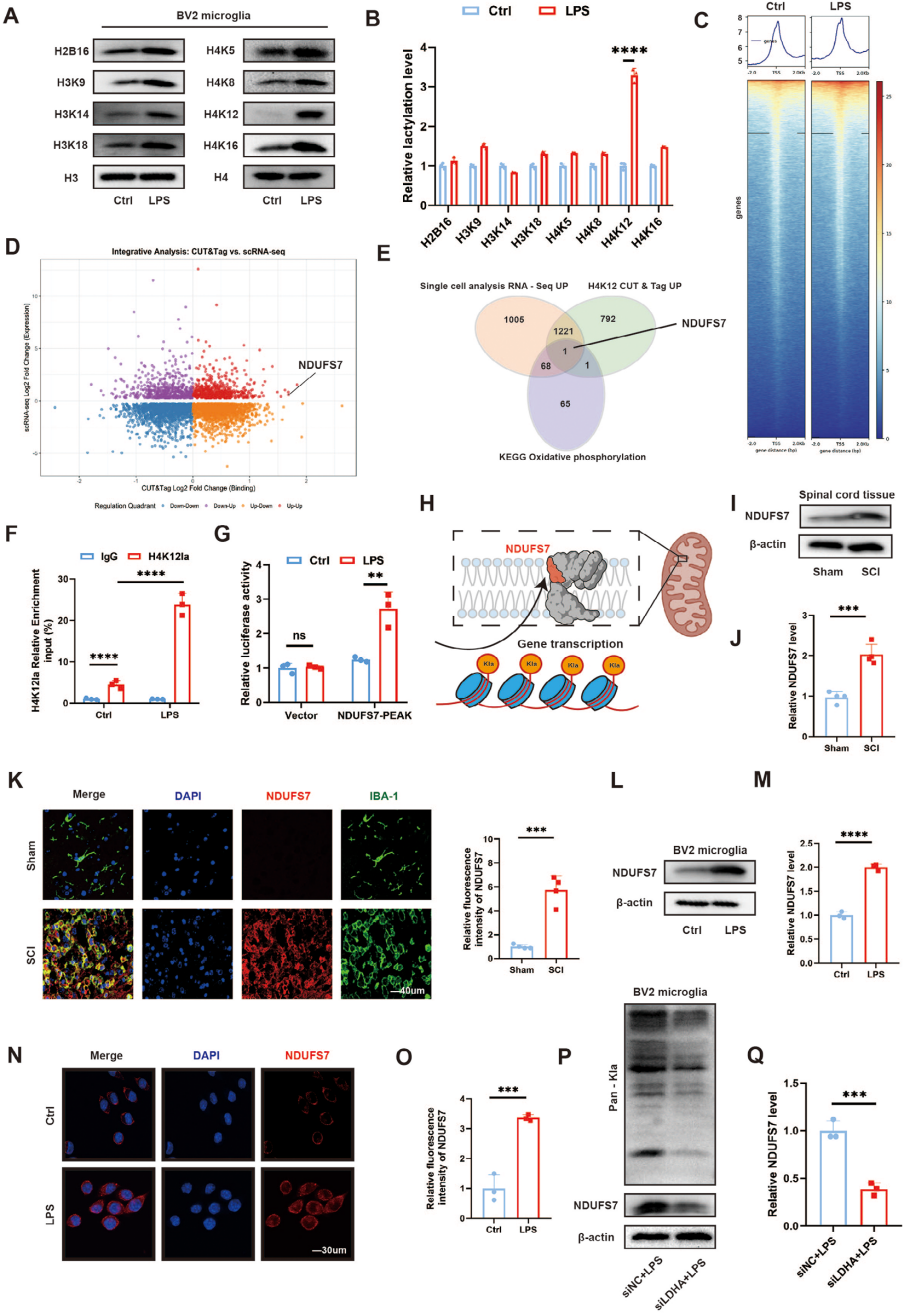
H4K12la directly targets and transcriptionally upregulates NDUFS7 in microglia. (A, B) Representative Western blot screening and quantification of eight specific histone lactylation sites in Ctrl and LPS‐stimulated BV2 microglia (*n* = 3). (C) Heatmap of CUT&Tag‐seq signals for H4K12la around transcription start sites (TSS) in Ctrl and LPS groups. (D) Integrative analysis scatter plot correlating gene expression changes (scRNA‐seq) with H4K12la binding intensity (CUT&Tag). (E) Venn diagram showing the intersection of scRNA‐seq upregulated genes, H4K12la CUT&Tag‐enriched peaks, and the KEGG Oxidative Phosphorylation pathway. (F) ChIP‐qPCR quantification of H4K12la enrichment at target regions (*n* = 3). (G) Dual‐luciferase reporter assay quantification of NDUFS7 activity (*n* = 3). (H) Schematic illustration of H4K12la‐mediated NDUFS7 transcription. (I, J) Representative Western blot bands and quantification of NDUFS7 expression in spinal cord tissues from Sham and SCI groups (*n* = 4). (K) Representative immunofluorescence images and analysis of NDUFS7 (red) and IBA‐1 (green) in spinal cord tissues (*n* = 4). (L, M) Western blot analysis and quantification of NDUFS7 levels in BV2 microglia (*n* = 3). (N, O) Representative immunofluorescence images and quantitative analysis of NDUFS7 expression in BV2 microglia (*n* = 3). (P, Q) Western blot analysis of Pan‐Kla and NDUFS7, and quantification of NDUFS7 in BV2 microglia transfected with siNC or siLDHA (*n* = 3). Data are presented as mean ± SD. Comparisons were performed using an unpaired *t*‐test (two groups) or two‐way ANOVA with Tukey's post hoc test as appropriate. ^*^ *p* < 0.05, ^**^ *p* < 0.01, ^***^ *p* < 0.001, and ^****^ *p* < 0.0001 vs. indicated group.

To further validate the specificity of H4K12la in microglia, we performed screening in primary microglial cultures. Consistent with our findings in BV2 microglia, Western blot analysis revealed that among the tested histone sites, H4K12la exhibited the most significant upregulation in microglia isolated after SCI (Figure S3A,B). Furthermore, we performed immunofluorescence co‐staining with cell‐specific markers. We observed a significant increase in H4K12la intensity specifically within IBA‐1^+^ microglia post‐SCI (Figure S3C,F). In contrast, no significant changes in H4K12la levels were detected in GFAP^+^ astrocytes or NeuN^+^ neurons (Figure S3D,E,G,H). These results confirm that H4K12la is predominantly enriched in activated microglia rather than other resident spinal cord cells following injury.

We next sought to identify the genomic targets of H4K12la. Cleavage Under Targets and Tagmentation (CUT&Tag) sequencing in microglia revealed a global enhancement of H4K12la occupancy across the genome after LPS treatment (Figure 2C). To identify the specific downstream targets driven by H4K12la, we performed a joint analysis of single‐cell RNA sequencing (scRNA‐seq) and CUT&Tag data (Figure 2D). By specifically focusing on the KEGG pathway related to mitochondrial oxidative phosphorylation, we successfully pinpointed NDUFS7 as a key intersection target (Figure 2E). Importantly, following the CUT&Tag analysis, independent ChIP‐qPCR assays confirmed a robust enrichment of H4K12la signals specifically at the identified NDUFS7 regulatory peak region (Figure 2F). To functionally validate this, a dual‐luciferase reporter assay was subsequently conducted. Consistent with the binding data, LPS stimulation significantly elevated the relative luciferase activity in cells transfected with the NDUFS7‐PEAK reporter plasmid, whereas no such induction was observed in the empty vector control group (Figure 2G). Together, these findings strongly demonstrate that this H4K12la‐enriched peak region possesses active, LPS‐inducible transcriptional regulatory activity, ultimately driving NDUFS7 expression. A schematic diagram further illustrates the proposed mechanism wherein enriched histone lactylation (Kla) at this regulatory peak region drives the expression of the mitochondrial subunit NDUFS7 (Figure 2H). This bioinformatic prediction was robustly validated in vivo, where western blot (Figure 2I,J) and immunofluorescence (Figure 2K) analyses confirmed a significant upregulation of NDUFS7 protein that strongly co‐localized with microglia in the injured spinal cord.

Consistently, in vitro experiments in BV2 microglia confirmed that LPS stimulation markedly increased NDUFS7 protein and mRNA levels (Figures 2L–O and S4A). Crucially, to establish a link between lactate metabolism, H4K12la, and NDUFS7 expression, we inhibited lactate production by transfecting cells with siLDHA. This intervention, which reduced global histone lactylation in LPS‐stimulated (Figure 2P), led to a significant decrease in NDUFS7 protein expression (Figure 2P,Q). Global lactylation, H4K12la, and NDUFS7 expression showed a positive correlation with lactate concentration (Figure S4B–E). These same markers exhibited a negative correlation with the concentration of the glycolysis inhibitor 2‐DG (Figure S4F–I). Taken together, these data demonstrate that H4K12 lactylation, driven by elevated lactate production during microglial activation, directly binds to the NDUFS7 gene locus and mediates its transcriptional upregulation.

To further validate the causal relationship between lactate metabolism, H4K12la, and NDUFS7 expression in vivo, we knocked down LDHA in the spinal cord of SCI mice using an AAV system. AAV‐shLDHA was injected 14 days before SCI, and spinal cord tissues were harvested at 28 Dpi (Figure S5A). Western blot showed that LDHA knockdown significantly reduced histone lactylation and H4K12la levels, and downregulated NDUFS7 protein expression (Figure S5B–F). Local lactate concentration at the injury site was also significantly decreased (Figure S5G).

### 2.3 Upregulation of NDUFS7 in Microglia Promotes Glycolytic Switch and Pro‐Inflammatory Microglia Polarization

To investigate the functional role of NDUFS7 in microglial polarization, we first selected siNDUFS7‐#2 (si#2), which exhibited the highest silencing efficacy using for subsequent experiments (Figure 3A,B). Knockdown of NDUFS7 in LPS‐stimulated microglia significantly attenuated the pro‐inflammatory phenotype, as evidenced by reduced immunofluorescence signal of the pro‐inflammatory marker CD86 (Figure 3C,D). Western blot analysis further confirmed that NDUFS7 silencing led to a marked decrease in pro‐inflammatory markers (iNOS, CD86) and a concurrent increase in anti‐inflammatory markers (Arg‐1, CD206) under LPS stimulation (Figure 3E,F).

**FIGURE 3:**
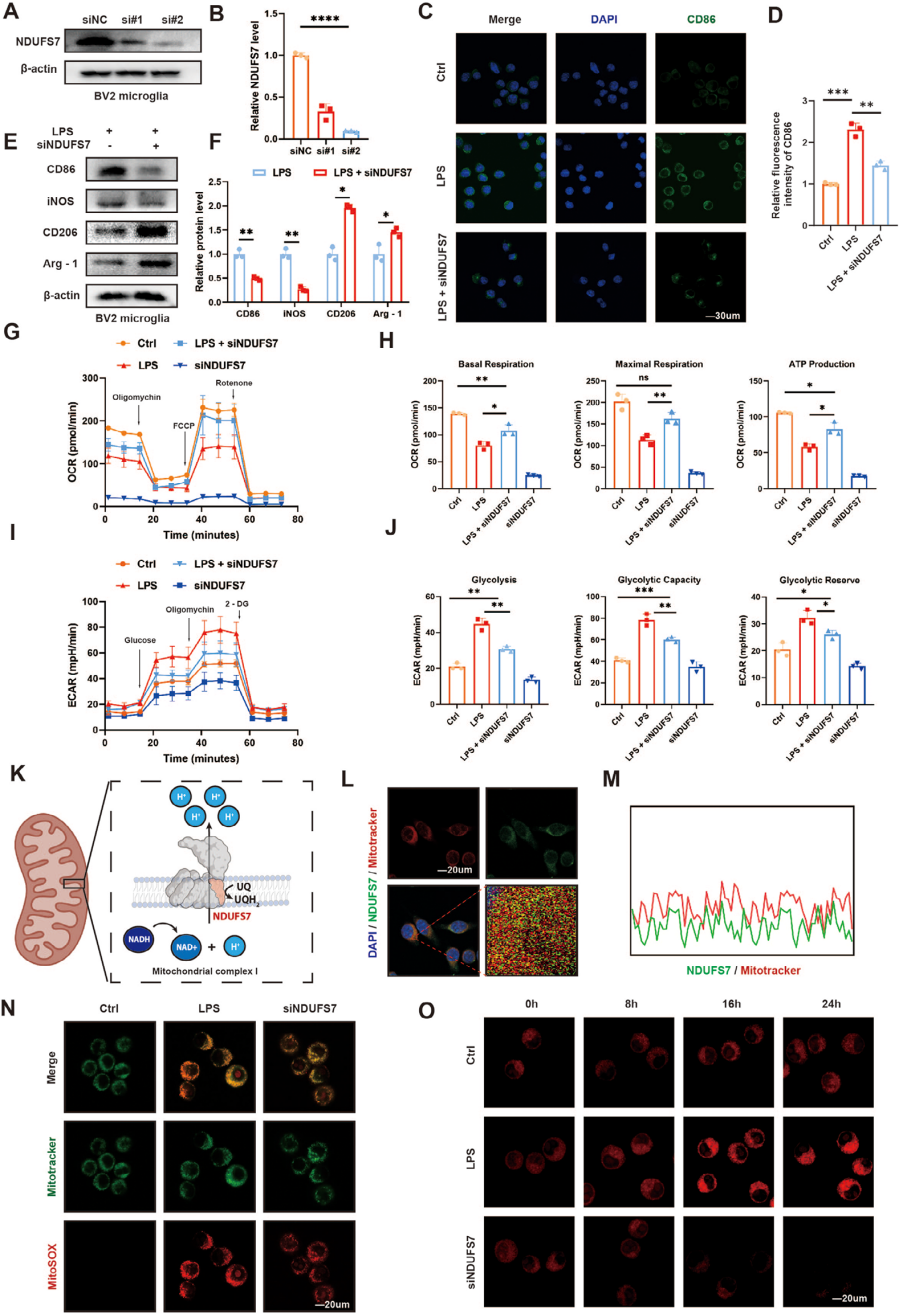
NDUFS7 is essential for LPS‐induced metabolic reprogramming and pro‐inflammatory polarization in microglia. (A, B) Western blot analysis and quantification of NDUFS7 knockdown efficiency using two siRNA sequences in BV2 microglia (*n* = 3). (C, D) Representative immunofluorescence images and quantitative analysis of CD86 (green) in Ctrl, LPS‐stimulated, and LPS + siNDUFS7‐treated cells (*n* = 3). (E, F) Representative Western blot bands and quantification of pro‐inflammatory (CD86, iNOS) and anti‐inflammatory (CD206, Arg‐1) polarization markers (*n* = 3). (G) Real‐time Oxygen Consumption Rate (OCR) measurements across different treatment groups (*n* = 3). (H) Quantification of basal respiration, maximal respiratory capacity, and ATP production (*n* = 3). (I) Real‐time Extracellular Acidification Rate (ECAR) measurements (*n* = 3). (J) Quantification of glycolysis, glycolytic capacity, and glycolytic reserve (*n* = 3). (K) Schematic diagram of electron transport and proton pumping at mitochondrial Complex I. (L, M) Representative confocal images and colocalization analysis of NDUFS7 (green) and MitoTracker (red). (N) Representative images of mitochondrial superoxide (MitoSOX, red) and mitochondria (MitoTracker, green) in Ctrl, LPS, and siNDUFS7 groups (n ≥ 4). (O) Time‐lapse imaging of mitochondrial membrane potential using TMRM staining over 24 h (*n* = 3). Data are presented as mean ± SD. Comparisons were performed using one‐way ANOVA or two‐way ANOVA with Tukey's post hoc test as appropriate. ^*^ *p* < 0.05, ^**^ *p* < 0.01, ^***^ *p* < 0.001, and ^****^ *p* < 0.0001 vs. indicated group.

Afterward, we assessed the impact of NDUFS7 on metabolic reprogramming. Seahorse XF analysis revealed that LPS stimulation induced a profound metabolic shift toward glycolysis, as indicated by a sharp increase in extracellular acidification rate and a decrease in oxygen consumption rate. Crucially, knockdown of NDUFS7 in LPS‐stimulated cells significantly mitigated this shift, partially restoring oxidative phosphorylation and attenuating glycolytic activity (Figure 3G–J). Of note, BV2 microglia with siNDUFS7 exhibited the lowest metabolic activity in both OCR and ECAR assays, suggesting a fundamental role for NDUFS7 in maintaining basal microglial bioenergetics (Figure 3G–J).

Given that NDUFS7 is a core subunit of mitochondrial Complex I, which is critical for NADH oxidation and proton pumping (Figure 3K) and confirmed to localize within mitochondria (Figure 3L,M), we further investigated its role in mitochondrial integrity and mtROS production. Interestingly, while both LPS stimulation and NDUFS7 knockdown increased mitochondrial superoxide production (MitoSOX) (Figures 3N and S6A), their effects on the mitochondrial membrane potential (ΔΨm) were fundamentally divergent. LPS treatment resulted in a hyperpolarized ΔΨm, as indicated by an increased JC‐1 aggregate‐to‐monomer ratio (Figure S6B,C) and a time‐dependent enhancement in TMRM (Tetramethylrhodamine Methylester) fluorescence (Figures 3O and S6D). This state of high proton‐motive force coupled with elevated mtROS is a classic prerequisite for driving reverse electron transport (RET) at Complex I.

In contrast, NDUFS7 knockdown alone led to a severe loss of ΔΨm (Figures 3O and S6D). This implies that destabilizing this core Complex I subunit directly causes forward electron leakage to oxygen (generating MitoSOX signals), while the impaired proton‐pumping function of the dysfunctional complex results in the concomitant collapse of ΔΨm. Collectively, these findings establish that H4K12la‐induced upregulation of NDUFS7 acts as a critical driver of the metabolic switch toward glycolysis, mitochondrial dysfunction, and the pro‐inflammatory polarization of microglia.

### 2.4 Elevated NDUFS7 Triggers RET and mtROS Burst via Complex I Hyperactivity

Overexpression of NDUFS7, like LPS stimulation, significantly increased mtROS levels. However, when the mitochondrial membrane potential was reduced by FCCP (carbonyl cyanide‐4‐(trifluoromethoxy) phenylhydrazone), which produced pathological mtROS, neither NDUFS7 overexpression nor LPS treatment could induce mtROS production (Figure S7A,B). Given that a hyperpolarized membrane potential is a strict thermodynamic prerequisite for driving RET, these results strongly indicate that the oxidative burst induced by NDUFS7 overexpression or LPS is specifically RET‐derived. We interpret NDUFS7 overexpression as driving mitochondrial Complex I into a hyperactive state, which aligns with prior reports indicating that enhanced Complex I activity sustains RET occurrence [9].

Subsequently, we investigated the relationship between NDUFS7 and RET, which produced pathological mtROS. As expected, ATP levels were the highest in control cells (Figure 4A,B). LPS stimulation, rotenone (Rot, a Complex I inhibitor) [34], or NDUFS7 knockdown, when applied alone, significantly reduced ATP levels (Figure 4B). Intriguingly, both Rot and siNDUFS7 treatment in the context of LPS stimulation resulted in a partial but significant recovery of ATP levels (Figure 4B), suggesting that inhibiting hyperactive Complex I under inflammatory conditions restores energetic balance. Consistent with the ATP levels, both NDUFS7 knockdown and Rot treatment after LPS stimulation effectively scavenge mtROS (Figure 4C,D) that upregulation of NDUFS7 following LPS stimulation leads to RET, while inhibition of NDUFS7 expression under LPS could suppress RET occurrence. Furthermore, flow cytometry analysis revealed that total cellular ROS was induced by LPS, which was suppressed by Complex I inhibition (Figure 4E).

**FIGURE 4:**
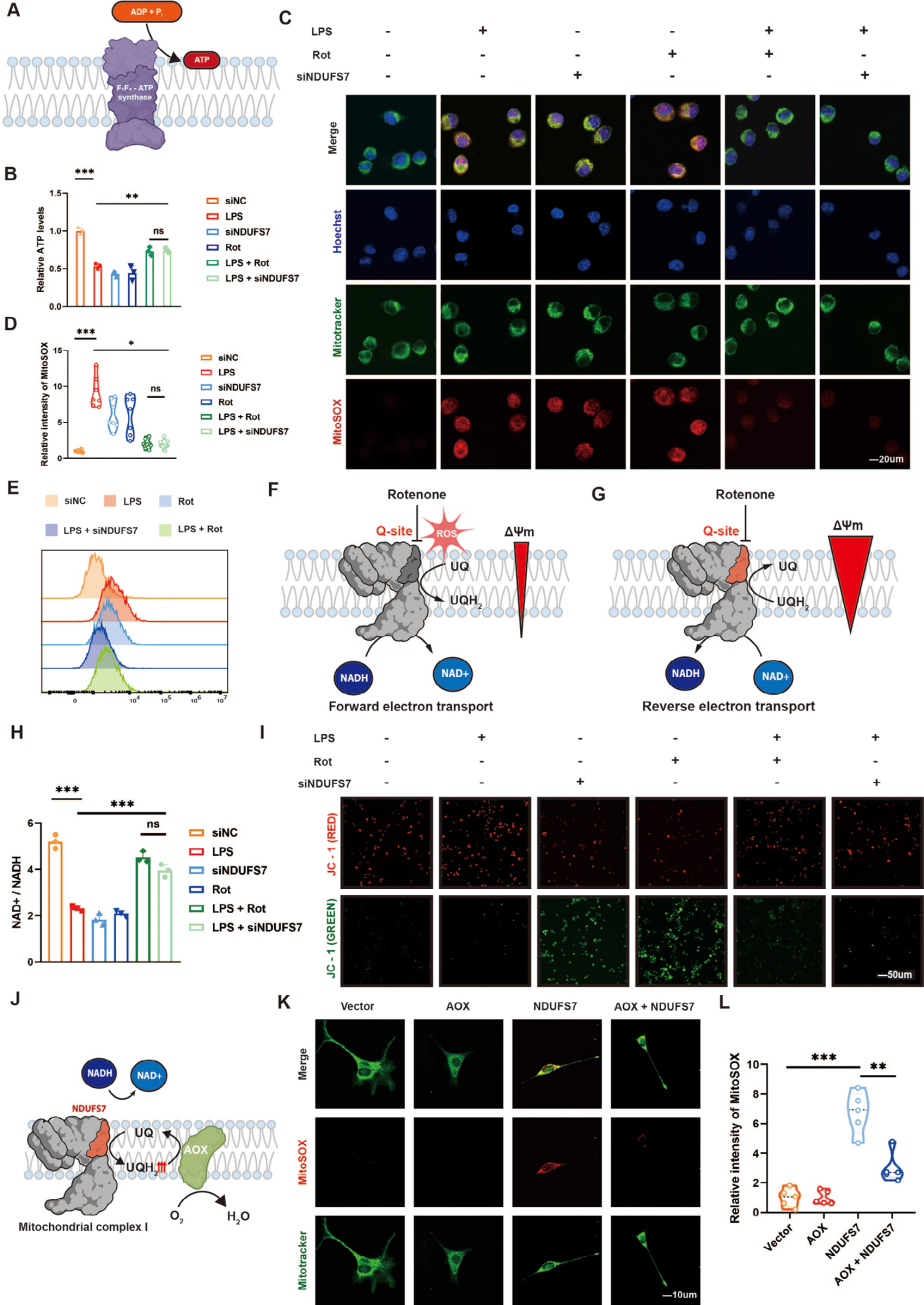
NDUFS7‐mediated mitochondrial ROS generation depends on RET at Complex I. (A) Schematic of mitochondrial ATP synthesis by F1Fo‐ATP synthase. (B) Measurement of intracellular ATP levels across different treatment groups (siNC, LPS, siNDUFS7, Rot, LPS + Rot, LPS + siNDUFS7) (*n* = 3). (C, D) Representative MitoSOX staining images and quantitative analysis of mitochondrial ROS (n ≥ 4). (E) Flow cytometry analysis of ROS levels across the indicated treatment groups (*n* = 3). (F, G) Schematic diagrams of Rotenone inhibition during Forward Electron Transport and Reverse Electron Transport. (H) Quantification of the NAD^+^/NADH ratio (*n* = 3). (I) Representative JC‐1 staining images. Red: JC‐1 aggregates (high ΔΨm); Green: JC‐1 monomers (low ΔΨm) (*n* = 3). (J) Schematic mechanism of Alternative Oxidase (AOX). (K, L) Representative MitoSOX images and quantification in HEK293T cells transfected with Vector, AOX, NDUFS7, or AOX + NDUFS7 (*n* ≥ 4). Data are presented as mean ± SD. Comparisons were performed using one‐way ANOVA with Tukey's post hoc test as appropriate. ^*^ *p* < 0.05, ^**^ *p* < 0.01, ^***^ *p* < 0.001, and ^****^ *p* < 0.0001 vs. indicated group.

During FET, electrons reach the Q‐site of Complex I; rotenone occupies this site, halting electron flow and proton pumping, thereby driving upstream over‐reduction and increased mtROS production (Figure 4F) [35]. Under high Δψm and a highly reduced CoQ pool, electrons flow backward via the Q‐site; rotenone blocks this site, halting the backflow and rapidly suppressing RET‐derived mtROS (Figure 4G) [34]. As an essential component of the Q site, inhibiting NDUFS7 expression produces an effect similar to that of rotenone. The LPS‐induced redox imbalance was further confirmed by a significant drop in the NAD^+^/NADH ratio, which was also partially rescued by siNDUFS7 and Rot treatment (Figure 4H). The sustained hyperpolarization of the mitochondrial membrane potential induced by LPS was reversed by both rotenone and siNDUFS7(Figures 4I and S7C), the flow cytometry results based on JC‐1 staining showed a consistent outcome (Figure S7D). AOX provides an alternative pathway by directly accepting electrons from the overloaded CoQH_2_ pool and transferring them to oxygen to form water. This rapid oxidation decreases the CoQH_2_/CoQ ratio, ultimately preventing mtROS generation via RET (Figure 4J) [36], so we transfected AOX and overexpressed NDUFS7 into HEK293T cell lines to confirm that NDUFS7 drives mtROS production specifically through RET (Figure 4K,L). Collectively, these data identify NDUFS7 upregulation as a key driver of the inflammatory mitochondrial ROS burst by promoting the conditions for RET.

### 2.5 Inhibiting RET Blocks Pro‐Inflammatory Microglia Polarization

To establish a causal link between RET and microglial polarization, we adopted a strategy using succinate treatment to create conditions favorable for RET induction [15, 37]. With the addition of succinate following LPS stimulation, the mitochondrial membrane potential remained elevated (Figure 5A,B). Succinate potentiates LPS‐induced mitochondrial mtROS production (Figure 5C,D). LPS stimulation significantly reduced ATP levels and the NAD^+^/NADH ratio; succinate can exacerbate its severity (Figure 5E,F). Cumulative evidence indicates that succinate exacerbates RET, which is consistent with previous research [8, 15]. We propose that in microglia, RET is also associated with their polarization status and influences their inflammatory secretory profile.

**FIGURE 5:**
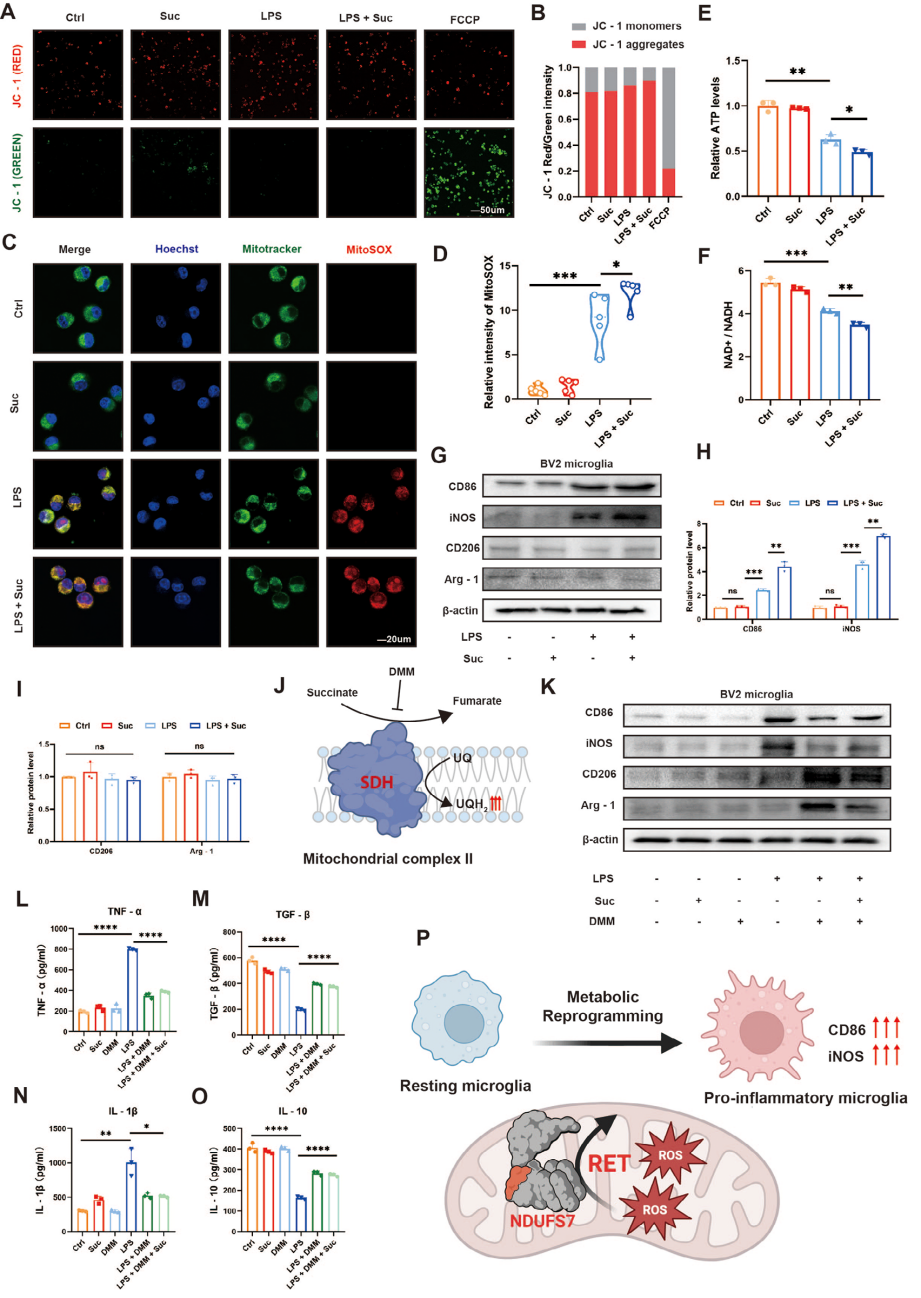
RET is a critical checkpoint driving pro‐inflammatory microglial polarization. (A, B) Representative JC‐1 staining images and quantification of the red/green fluorescence ratio across the indicated treatment groups (*n* = 3). (C, D) Representative MitoSOX staining images and quantitative analysis of mitochondrial ROS levels (*n* ≥ 4). (E, F) Measurement of intracellular ATP levels and the NAD^+^/NADH ratio (*n* = 3). (G–I) Representative Western blot bands and quantification of pro‐inflammatory markers (CD86, iNOS) and anti‐inflammatory markers (CD206, Arg‐1) (*n* = 3). (J) Schematic diagram of dimethyl malonate (DMM) inhibition of the succinate dehydrogenase (SDH) complex. (K) Representative Western blot analysis of polarization markers in BV2 microglia under LPS, succinate, and DMM treatments (*n* = 3). (L–O) ELISA analysis of the inflammatory cytokines TNF‐α, TGF‐β, IL‐1β, and IL‐10 in cell culture supernatants (*n* = 3). (P) Schematic model of succinate‐driven RET, mitochondrial ROS burst, and pro‐inflammatory microglial polarization mediated by NDUFS7. Data are presented as mean ± SD. Comparisons were performed using one‐way ANOVA with Tukey's post hoc test as appropriate. ^*^ *p* < 0.05, ^**^ *p* < 0.01, ^***^ *p* < 0.001, and ^****^ *p* < 0.0001 vs. indicated group.

To directly link RET to microglial polarization, we profiled classic pro‐inflammatory microglia/anti‐inflammatory microglia. Western blot analysis showed that succinate treatment alone had a minimal effect on polarization markers but potently synergized with LPS to enhance the expression of pro‐inflammatory markers (CD86, iNOS) and had no significant impact on anti‐inflammatory markers (CD206, Arg‐1) (Figure 5G–I). Crucially, this pro‐inflammatory effect was abrogated by Dimethyl malonate (DMM), an inhibitor of the succinate dehydrogenase (SDH) complex that blocks the oxidation of succinate to fumarate (Figure 5J) [15, 38], which effectively restored a more balanced polarization state even in the presence of LPS (Figures 5K and S8A,B), corresponding pro‐ and anti‐inflammatory levels (Figures 5L–O and S8C–F). Collectively, these data demonstrate that driving RET serves as a potent amplifier of the pro‐inflammatory microglial response. Together with our previous findings, this establishes RET as a convergent metabolic switch governing microglial fate, regardless of H4K12la‐induced NDUFS7 overexpression or succinate accumulation (Figure 5P).

To extend these findings in vivo, we assessed microglial polarization and mitochondrial RET status in SCI mice after LDHA knockdown. AAV‐shLDHA significantly reduced CD86 and DHE fluorescence in spinal cord microglia (Figure S9A–D), and decreased mtROS in primary microglia (Figure S9E,F). Notably, LDHA silencing restored NAD^+^/NADH ratios and ATP levels (Figure S9G,H) alongside reduced lactate (Figure S9I). These results indicate that LDHA‐driven lactate metabolism aggravates RET to promote pro‐inflammatory microglial polarization in vivo.

### 2.6 Fabrication of Microglial Membrane‐Coated ROS‐Responsive Micelles for PROTAC Therapy

To design a PROTAC molecule targeting LDHA for degradation (Figure 6A). The workflow for screening LDHA affinity ligands as PROTAC warheads is illustrated in Figure S10A. We first screened the ChEMBL database for warhead candidates with high affinity to LDHA. A total of 1,125 LDHA binders were identified (Figure S10B). From these, candidates with an IC50< 10 nm were selected, yielding 16 compounds; each of these was subjected to molecular docking analysis (Figure S10C). The compounds CHEMBL4520899, CHEMBL5176448, and CHEMBL4475593 achieved the top three highest RDKit scores (Figure S10D). After further evaluation based on IC50 values, the ligand with the lowest (IC50 = 2.9 nm), CHEMBL5176448, was chosen. Its molecular docking profile and chemical structure are presented (Figure 6B,C). This selected warhead was then conjugated with an appropriate linker and an E3 ligase ligand to synthesize the final PROTAC molecule (Figure 6D). The optimal concentration of the PROTAC was tested in BV2 microglia, revealing that 1 µm had no effect on cell proliferation while exhibiting high degradation efficiency (Figures 6E,F and S10E).

**FIGURE 6:**
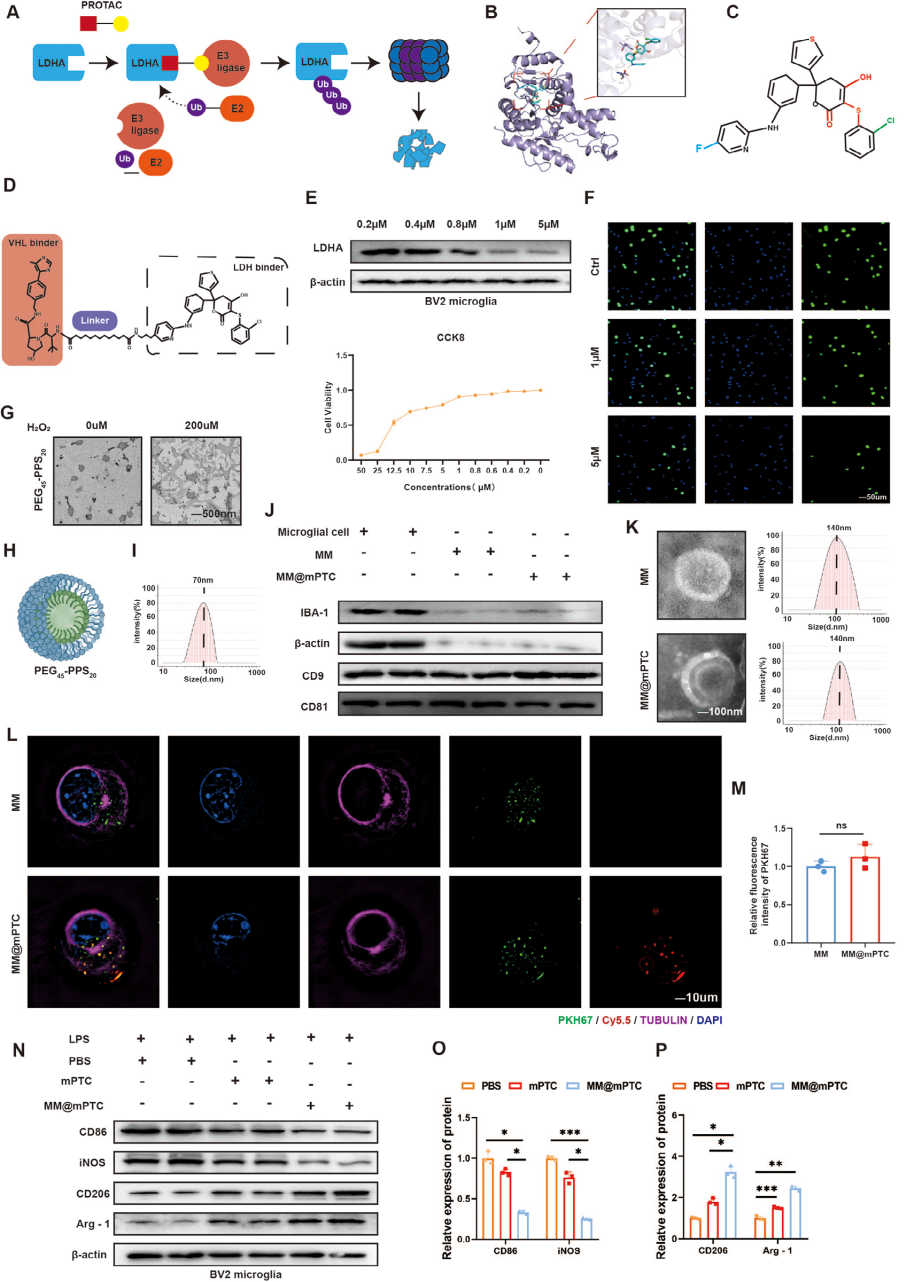
Engineering and characterization of the ROS‐responsive biomimetic PROTAC delivery system (MM@mPTC). (A) Schematic of the PROTAC mechanism for targeted LDHA degradation. (B) Molecular docking simulation of the LDHA ligand (CHEMBL5176448) with the LDHA protein. (C) Chemical structure of the LDHA‐targeting ligand, CHEMBL5176448. (D) Chemical structure of the synthesized LDHA‐targeting PROTAC. (E) Western blot analysis of LDHA degradation (upper panel) and CCK‐8 cell viability assay (lower panel) under varying PROTAC concentrations (*n* = 3). (F) Representative EdU staining images of cell proliferation (*n* = 3). (G) Representative TEM images of PEG45‐PPS20 micelles treated with 0 µm or 200 µm H_2_O_2_ (*n* = 3). (H, I) Schematic diagram and particle size distribution profile of the PEG45‐PPS20 micelles (*n* = 3). (J) Western blot characterization of extracted microglial cell membranes (MM) and membrane‐coated biomimetic nanoparticles (MM@mPTC) for membrane (CD9, CD81) and cytosolic (IBA‐1, β‐actin) markers (*n* = 3). (K) Representative TEM images (left) and size distribution profiles (right) of pure MM and MM@mPTC (*n* = 3). (L, M) Representative confocal fluorescence images and quantification of cellular uptake of PKH67‐labeled MM and intracellular delivery of PROTAC‐Cy5.5 in BV2 microglia (*n* = 3). (N) Representative Western blot analysis of polarization markers in LPS‐stimulated BV2 microglia treated with PBS, mPTC, or MM@mPTC (*n* = 3). (O–P) Quantification of pro‐inflammatory (CD86, iNOS) and anti‐inflammatory (CD206, Arg‐1) polarization markers (*n* = 3). Data are presented as mean ± SD. Comparisons were performed using an unpaired *t*‐test (two groups), or two‐way ANOVA with Tukey's post hoc test, as appropriate. ^*^ *p* < 0.05, ^**^ *p* < 0.01, ^***^ *p* < 0.001, and ^****^ *p* < 0.0001 vs. indicated group.

After that, our group engineered a ROS‐responsive micelle. We synthesized PEG_45_‐PPS_20_ polymeric micelles, which were verified by TEM to demonstrate their H_2_O_2_‐triggered disassembly and payload release (Figure 6G). The successful synthesis and chemical structure of the PEG_45_‐PPS_20_ copolymer were first confirmed by ^1^H NMR spectroscopy (Figure S10F). A schematic diagram of the PEG_45_‐PPS_20_ micelle is shown in Figure 6H. These blank micelles exhibited a uniform size of approximately 70 nm (Figure 6I). The LDHA‐targeting PROTAC was then encapsulated within these micelles, forming the core nanoparticle (mPTC). To further enhance microglia‐specific targeting, we extracted microglial cell membranes (MM) for biomimetic coating. Western blot analysis confirmed the successful isolation and high purity of the biomimetic membranes, as evidenced by the significant enrichment of membrane markers (CD81, CD9), alongside the absence of the intracellular cytosolic protein IBA‐1 and β‐actin (Figure 6J). Subsequently, we used microglial membranes to load the mPTC nanoparticles. The resulting biomimetic nanoparticles (MM@mPTC) displayed a spherical core‐shell morphology indicative of successful membrane cloaking, with a particle size of approximately 140 nm (Figure 6K). Furthermore, confocal microscopy confirmed the efficient cellular uptake of these membrane‐coated PROTAC by BV2 microglia in vitro (Figure 6L,M). Finally, we validated the therapeutic efficacy of MM@mPTC in vitro. Treatment of LPS‐stimulated BV2 microglia with MM@mPTC effectively reversed the pro‐inflammatory polarization, as demonstrated by a significant decrease in pro‐inflammatory markers and an increase in anti‐inflammatory markers (Figure 6N–P). Notably, the MM@mPTC intervention significantly accelerated PC12 cell migration, exhibiting markedly faster wound closure at 24 h compared to the MM‐treated and mPTC‐treated groups under LPS stimulation (Figure S10G). Collectively, we have successfully fabricated a sophisticated, multi‐layered nanotherapeutic that combines targeted LDHA degradation, ROS‐responsive release, and microglia‐specific delivery. Moreover, MM@mPTC showed a potent anti‐inflammatory effect in cultured BV2 microglia.

### 2.7 MM@mPTC Promotes Spinal Cord Preservation and Axonal Regeneration after Spinal Cord Injury

Subsequently, we evaluated the in vivo targeting capability and biodistribution of our nanotherapeutics in a mouse model of SCI. The treatment timeline is schematized in Figure 7A. To enable fluorescence tracking, mice were intravenously administered PBS, PROTAC‐Cy5.5, or the equivalent formulations encapsulated in mPTC and MM@mPTC immediately following SCI (0 Dpi). In vivo IVIS imaging confirmed the targeted accumulation at the injury site; the MM@mPTC group exhibited the most intense fluorescence signal at the lesion compared to all other groups, demonstrating its superior targeting and retention capability at 7 Dpi (Figure 7B). At 28 Dpi, IVIS imaging of the ex vivo spinal cords confirmed the highest retention of the MM@mPTC at the lesion site (Figure 7C). Imaging of major organs (heart, liver, spleen, lung, and kidney) revealed predominant accumulation in the liver at 28 Dpi, suggesting hepatic clearance as the main elimination pathway (Figure 7D). Furthermore, histological analysis (H&E staining) of major organs revealed no apparent pathological lesions, confirming the good biocompatibility of all treatments (Figure S11A).

**FIGURE 7:**
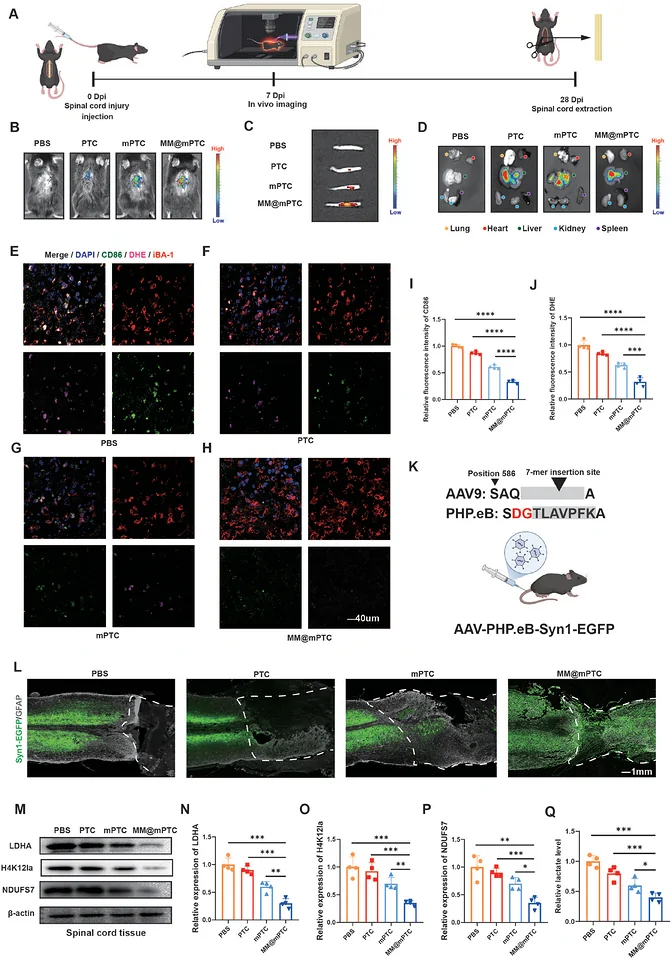
MM@mPTC targets the injured spinal cord to alleviate inflammation and promote tissue repair in vivo. (A) Schematic illustration of the in vivo experimental workflow, including SCI modeling, drug administration, and tissue harvesting timeline. (B) Representative in vivo fluorescence imaging of mice at 7 Dpi (*n*  ≥  4). (C, D) Ex vivo fluorescence imaging of spinal cord tissues and major organs at 28 Dpi (n  ≥  4). (E, H) Representative immunofluorescence staining of CD86 (green), DHE (purple), and IBA‐1 (red) (*n* = 4). (I, J) Quantitative analysis of CD86 and DHE fluorescence intensity (*n* = 4). (K) Illustrates the adeno‐associated virus vector used for specific neuronal labeling, featuring a 7‐mer insertion site in the PHP.eB capsid protein and EGFP expression driven by the Syn1 promoter. (L) Representative immunofluorescence staining of Syn1‐EGFP (green) and GFAP (gray) (n ≥ 4). (M) Western blot detection of LDHA, H4K12la, and NDUFS7 protein levels in spinal cord tissues (*n* = 4). (N, Q) Quantitative analysis of the relative expression levels of LDHA, H4K12la, NDUFS7, and lactate (*n* = 4). Data are presented as mean ± SD. Comparisons were performed using one‐way ANOVA with Tukey's post hoc test as appropriate. ^*^ *p* < 0.05, ^**^ *p* < 0.01, ^***^ *p* < 0.001, and ^****^ *p* < 0.0001 vs. indicated group.

We further assessed the impact of MM@mPTC on key pathological hallmarks of SCI at 28 Dpi. Immunofluorescence staining of spinal cord sections demonstrated that MM@mPTC treatment most effectively reduced the levels of oxidative stress (DHE) and the abundance of CD86^+^/IBA‐1^+^ pro‐inflammatory microglia within the lesion (Figure 7E–J). Furthermore, AAV‐PHP.eB‐Syn1‐EGFP tracing demonstrated robust axonal regeneration in the MM@mPTC‐treated group (Figure 7K,L). Consistently, NF200/GFAP co‐staining revealed significantly elevated NF200 fluorescence intensity and markedly reduced GFAP immunoreactivity, reflecting enhanced neuronal/axonal preservation and suppressed astrogliosis in the MM@mPTC group (Figure S11B–D). Finally, histological evaluations through H&E, Masson, and Nissl staining collectively confirmed that MM@mPTC treatment resulted in the most significant mitigation of tissue damage, cavity formation, and collagen deposition, alongside the best‐preserved neural tissue (Figure S11E). Western blot analyses showed that MM@mPTC treatment most effectively suppressed the LDHA‐H4K12la‐NDUFS7 axis in the injured spinal cord at 28 Dpi, significantly reducing LDHA expression, H4K12 lactylation, NDUFS7 levels, and tissue lactate content compared with PBS and PTC controls (Figure 7M–Q). We isolated primary microglia at 28 Dpi after treatment; MM@mPTC treatment significantly decreased the MitoSOX relative intensity compared to other groups (Figure S12A,B). Additionally, MM@mPTC notably enhanced the NAD^+^/NADH ratio, as well as the relative ATP, and decreased the lactate level (Figure S12C–E). Collectively, these results indicate that MM@mPTC effectively blocks RET after SCI. NeuN immunofluorescence staining further revealed distinct differences in neuronal preservation across treatment groups. Spatial analysis across zones Z1‐Z4 showed that neuronal sparing in the PTC groups was largely restricted to Z 43‐Z 34. In the mPTC, NeuN^+^ neurons extended into the Z3‐Z2 region, while MM@mPTC preserved NeuN^+^ neurons up to the Z1 zone, closest to the lesion core (Figure S13A). In summary, these findings demonstrate that MM@mPTC efficiently targets the site of spinal cord injury, mitigates neuroinflammation and oxidative damage, and significantly promotes tissue preservation and repair.

### 2.8 MM@mPTC Improves Locomotor Recovery after Spinal Cord Injury

While the cellular mechanisms of recovery are crucial, the ultimate goal of SCI therapy is functional improvement. To obtain a precise and quantitative assessment of locomotor recovery at 28 Dpi, we employed video‐based kinematic analysis of hindlimb function. Key joints of the hindlimbs were marked for detailed motion tracking (Figure 8A). A concise overview of multiple kinematic parameters, presented in a radar chart, clearly demonstrated that the MM@mPTC treatment group exhibited a functional profile closest to the sham‐operated controls (Figure 8B). Although all injured mice showed some degree of movement, the MM@mPTC group displayed the most robust recovery, characterized by significantly improved anti‐gravity limb support during stepping.

**FIGURE 8:**
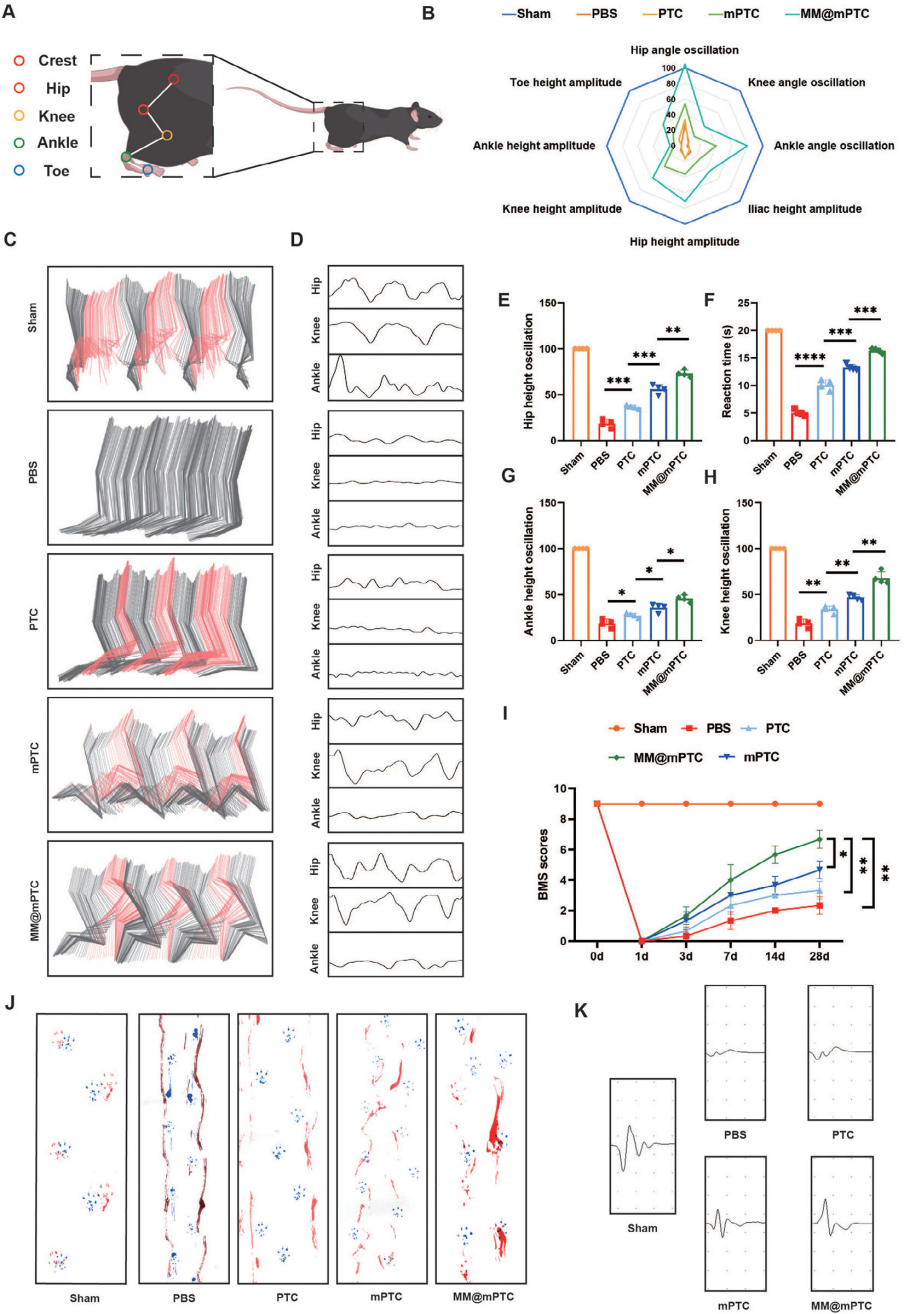
MM@mPTC promotes motor functional recovery and electrophysiological reconnections in SCI mice. (A) Schematic illustration of hindlimb gait motion, outlining the crest–hip–knee–ankle–toe movement trajectory used for kinematic analysis. (B) Radar plot summarizing the comprehensive functional parameters of each group at 28 Dpi (*n* = 4). (C) Representative hindlimb motion trajectories of mice in each group during a gait cycle at 28 Dpi. (*n* = 4). (D) Joint‐angle fluctuation curves of the hip, knee, and ankle during hindlimb movement at 28 Dpi (*n* = 4). (E–H) Quantitative analysis of the maximal vertical displacement for the Hip, the maximal reaction time, and the ankle, as well as the knee, at 28 Dpi (*n* = 4). (I) Basso Mouse Scale (BMS) scores evaluated from 0 to 28 Dpi (*n* = 4). (J) Representative gait footprints showing forelimb (blue) and hindlimb (red) step patterns at 28 Dpi (*n* = 4). (K) Representative MEP recordings from each group at 28 Dpi (*n* = 4). Data are presented as mean ± SD. Comparisons were performed using one‐way ANOVA with Tukey's post hoc test as appropriate. ^*^ *p* < 0.05, ^**^ *p* < 0.01, ^***^ *p* < 0.001, and ^****^ *p* < 0.0001 vs. indicated group.

Detailed kinematic analysis further revealed significant improvements in the MM@mPTC‐treated mice. This included a marked increase in the swing‐phase angles of the hip, knee, and ankle joints and reaction time, indicating a restoration of more normal and coordinated stepping patterns (Figure 8C–H). This refined locomotion was corroborated by established functional assays. The MM@mPTC group achieved the highest scores on the Basso Mouse Scale (BMS) for locomotor rating over the 28‐day observation period (Figure 8I). Footprint analysis visually confirmed the superior anti‐gravity capacity and stepping function in the MM@mPTC group (Figure 8J). Finally, electrophysiological recordings (MEPs) showed the most significant recovery of spinal cord conductivity in the MM@mPTC treated mice, providing physiological evidence for the restoration of neural transmission pathways (Figure 8K). Collectively, the data from quantitative kinematics, behavioral scoring, and electrophysiology provide convergent evidence that MM@mPTC treatment robustly promotes the recovery of coordinated hindlimb locomotion after spinal cord injury.

## 3 Discussion

Our work establishes that the LDHA‐lactate‐H4K12la signaling axis directly promotes NDUFS7 expression in pro‐inflammatory microglia. We identify this NDUFS7 upregulation as a critical molecular switch that initiates mitochondrial RET, driving the production of pathogenic mtROS and compromising recovery from spinal cord injury.

Recent evidence highlights the interplay between histone modifications and energy metabolism. Metabolites from the tricarboxylic acid cycle and glycolysis, such as acetyl‐CoA and lactate, serve as substrates for histone modifications [4, 39]. Metabolic reprogramming that increases the levels of these substrates may lead to significant alterations in histone modification profiles. As noted, primary mechanical trauma and subsequent vascular disruption following SCI create a hypoxic microenvironment [40]. In order to adapt to this bioenergetic crisis, reactive microglia undergo metabolic reprogramming toward anaerobic glycolysis [41], inevitably leading to the massive accumulation of lactate observed in the lesion site. Rather than acting solely as a metabolic waste product, this accumulated lactate serves as a critical epigenetic substrate. Elevated LDHA expression not only catalyzes lactate production but also expands the intracellular lactyl‐CoA pool [42], thereby directly fueling histone lactylation. Consequently, our findings demonstrate that this LDHA‐lactate‐histone lactylation axis is a key driver of pro‐inflammatory polarization in microglia, as evidenced by the fact that LDHA inhibition significantly abrogates both histone lactylation and pro‐inflammatory phenotypic markers. We acknowledge that histone lactylation is emerging as a critical epigenetic mechanism in SCI pathobiology, with different histone sites exerting distinct and cell‐type‐specific effects. In the context of spinal cord injury, H3K9la has been demonstrated to activate the TXNIP/NLRP3 axis, thereby exacerbating mitochondrial damage and amplifying inflammatory responses in macrophages [43]. Meanwhile, H4K8la has been shown to enhance astrocytic energy supply to neurons through a positive feedback loop, supporting neuronal survival under metabolic stress [44]. These findings illustrate that multiple histone lactylation sites differentially influence SCI outcomes through diverse cellular and molecular mechanisms. In exploring the specific epigenetic landscape, we conducted a systematic screening across a comprehensive panel of histone lactylation sites, including H2B16, H3K9, H3K14, H3K18, H4K5, H4K8, H4K12, and H4K16. Through this comparative analysis, H4K12la was identified as the predominant modification, exhibiting the most robust enrichment in the injury microenvironment. H4K12la is increasingly recognized for coordinating rapid metabolic and inflammatory responses through targeted gene activation [45, 46]. To map the direct functional target of this epigenetic mark, we performed an integrated analysis by intersecting oxidative phosphorylation‐related genes (KEGG), pro‐inflammatory microglia‐specific upregulated transcripts (scRNA‐seq), and H4K12la‐enriched regions (CUT&Tag). From this multi‐omics screening, NDUFS7 emerged as the sole overlapping target. Further CUT&Tag profiling confirmed that H4K12la directly occupies a specific regulatory peak region within the NDUFS7 locus to drive its transcription.

NDUFS7 functions as a structural anchor for the Complex I Q‐site, coordinating electron transfer at the terminal N2 [4Fe–4S] cluster [16, 34, 47]. It might be intuitive to assume that increased NDUFS7 expression would simply optimize respiratory chain efficiency. Our data suggest a pathological consequence: the abundance of functional Q‐sites drives excessive proton‐pumping capacity. We validated this biophysical alteration through TMRM and JC‐1 staining, which revealed that LPS‐induced microglia exhibit sustained hyperpolarization of the inner mitochondrial membrane. From a bioenergetic perspective, this persistently high membrane potential creates a thermodynamic barrier that impedes forward electron transport [48, 49]. Instead, the high electrochemical gradient forces electrons to flow backward from the reduced ubiquinone pool into Complex I—the defining characteristic of RET [12]. In this state, electrons prematurely reduce molecular oxygen at upstream iron‐sulfur clusters, generating a substantial mtROS burst [11]. Given that NDUFS7 is a critical core subunit governing the electron transfer dynamics within the ubiquinone‐binding module, we sought to verify that this ROS surge is indeed a direct consequence of the NDUFS7‐mediated RET pathway. Using a panel of interventions, we found the mtROS burst was markedly blunted by rotenone and DMM. Crucially, silencing NDUFS7 reproduced this effect. Furthermore, the specific introduction of Alternative Oxidase (AOX)—which dissipates the reduced CoQ pool—effectively abrogated the NDUFS7‐induced ROS, providing direct evidence that NDUFS7 drives oxidative stress strictly via the RET pathway.

To decisively establish the causal link between this RET phenomenon and microglial fate determination, we extended our investigation using succinate—a substrate whose oxidation drives the direct reduction of the ubiquinone pool, creating ideal thermodynamic conditions for RET induction [15]. We observed that succinate supplementation significantly potentiated LPS‐induced bioenergetic stress, exacerbating the mtROS burst and sustaining high mitochondrial membrane potential, while concurrently depleting ATP levels and reducing the NAD^+^/NADH ratio. Phenotypically, this metabolic state acted as a potent amplifier of neuroinflammation: succinate synergized with LPS to robustly upregulate pro‐inflammatory markers (CD86, iNOS) and suppress anti‐inflammatory markers (CD206, Arg‐1). Crucially, this pro‐inflammatory shift was strictly dependent on RET activity, as the phenotype was effectively rescued by dimethyl malonate (DMM), an inhibitor that blocks succinate oxidation and cuts off the electron supply for RET [15, 38]. These findings identify RET as a convergent metabolic switch governing microglial polarization. By utilizing succinate to mechanistically recapitulate a high‐RET state, we confirmed that driving electron reversal is sufficient to amplify the pro‐inflammatory response. Crucially, this validation establishes the H4K12la‐mediated upregulation of NDUFS7 not merely as a correlate, but as the specific pathological trigger that chronically engages this RET machinery to lock microglia in a neurotoxic pro‐inflammatory state following spinal cord injury.

Finally, to translate these mechanistic insights into a targeted therapeutic intervention, we employed a computer‐aided rational design strategy to engineer a biomimetic nanoplatform, MM@mPTC. Addressing the upstream metabolic trigger, we performed a comprehensive in silico screening of the ChEMBL database, combining high‐affinity filtering with molecular docking to identify the optimal warhead (CHEMBL5176448) for constructing a specific LDHA‐degrading PROTAC [50]. To ensure precise delivery of this payload to the inflamed microenvironment, we leveraged the pathological signature of pro‐inflammatory microglia—specifically, the RET‐induced oxidative stress. We synthesized a ROS‐responsive PEG_45_‐PPS_20_ copolymer micelle, where the hydrophobic PPS core serves as an intelligent sensor that undergoes phase transition upon oxidation, triggering on‐demand drug release [51]. To further enhance targeting specificity, this core was camouflaged with MM, endowing the biomimetic system with natural biological tropism [28, 52, 53]. Functionally, MM@mPTC operates through a coordinated cascade: it specifically degrades LDHA to cut off the lactate supply, thereby preventing H4K12 lactylation and the subsequent NDUFS7 upregulation (Figure 9). This intervention effectively dismantles the thermodynamic machinery required for RET, quenching the mtROS burst at its source and facilitating the repolarization of microglia. Both in vitro and in vivo studies demonstrated high targeting efficiency, significant anti‐inflammatory and neuroprotective effects, effective reversal of pro‐inflammatory microglial polarization, and‐most notably‐substantial promotion of locomotor recovery in mice with spinal cord injury. This integrated strategy represents a promising and precise therapeutic approach for spinal cord injury and other neuroinflammatory diseases.

**FIGURE 9:**
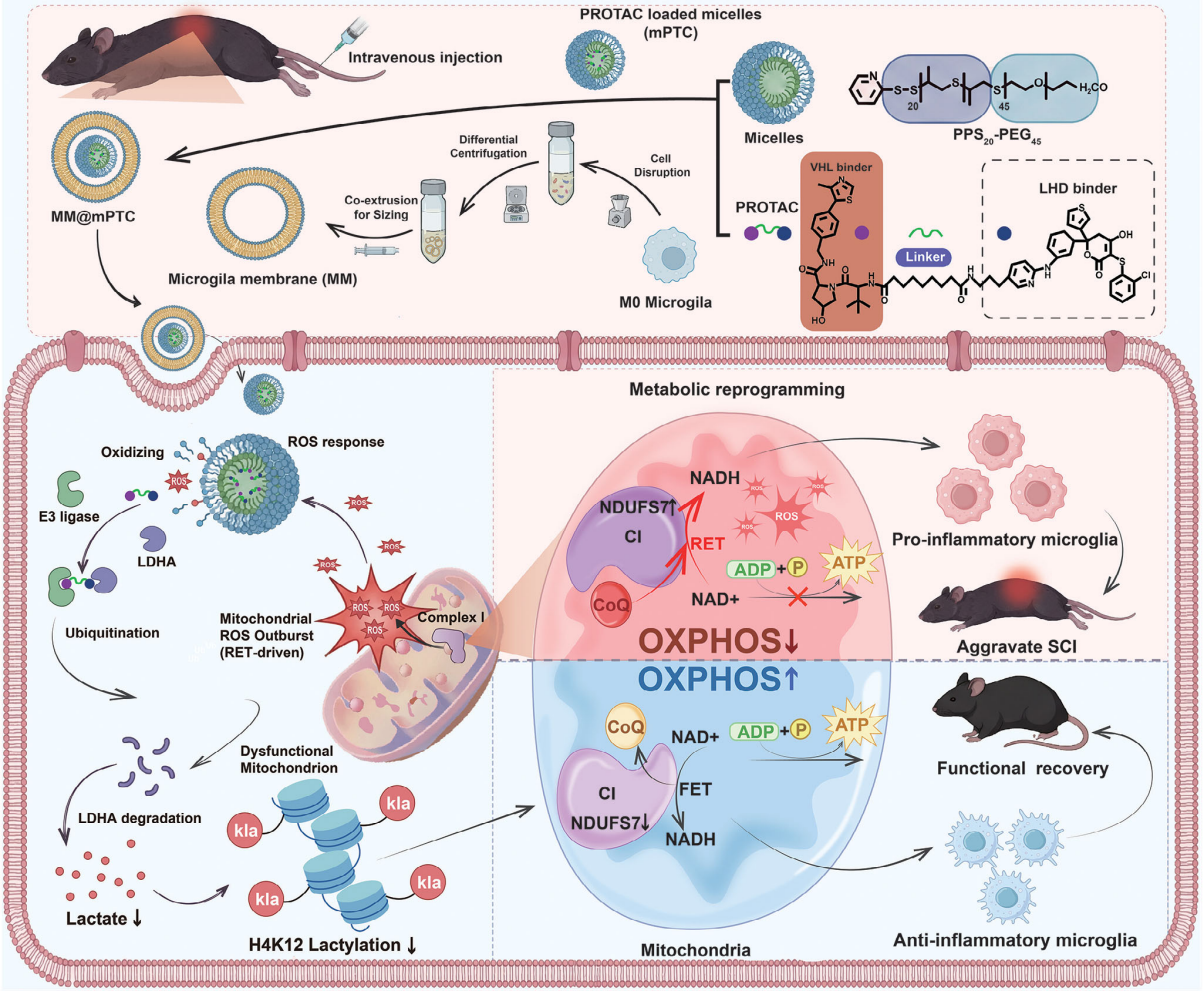
Schematic illustration of H4K12 lactylation driving NDUFS7 transcription to trigger RET, and the therapeutic mechanism of MM@mPTC. Pathological RET generates a mitochondrial ROS outburst that specifically triggers the ROS‐responsive MM@mPTC to release its PROTAC payload. The resulting PROTAC‐mediated LDHA degradation lowers cellular lactate levels, which in turn decreases H4K12 lactylation. This epigenetic reduction downregulates NDUFS7 expression, ultimately shutting down the pathological RET cycle and reprogramming microglia to promote functional recovery.

## 4 Conclusion

In summary, our study demonstrates that lactate‐driven H4K12 lactylation upregulates NDUFS7. This modification acts as a key switch that maintains microglia in a pro‐inflammatory state through continuous RET‐induced oxidative stress. Furthermore, by using MM@mPTC to block this LDHA‐H4K12la‐NDUFS7‐RET cascade, we not only elucidate a fundamental mechanism of microglial polarization but also offer a promising therapeutic strategy for treating spinal cord injury and broader neuroinflammatory diseases.

## 5 Methods

### 5.1 scRNA‐seq Data Analysis

Single‐cell RNA sequencing (scRNA‐seq) data of the mouse spinal cord were obtained from the GEO database (GSE234774). Raw count matrices were imported and processed using the Seurat R package. Following standard quality control to filter out low‐quality cells, the data underwent normalization, scaling, and Principal Component Analysis. Uniform Manifold Approximation and Projection (UMAP) was utilized for non‐linear dimensionality reduction and visualization. Cell clusters were identified and annotated using canonical marker genes, enabling the specific extraction of the microglia/macrophage population for downstream analysis. Differentially expressed genes (DEGs) between conditions were identified using the FindMarkers function (adjusted *p* < 0.05). Subsequently, GO and KEGG pathway enrichment analyses were performed via the clusterProfiler package to elucidate the metabolic reprogramming profiles, specifically focusing on targets such as NDUFS7.

### 5.2 Cleavage under Targets and Tagmentation (CUT&Tag) Assay

To profile the genome‐wide distribution of histone H4 lysine 12 lactylation (H4K12la), the CUT&Tag assay was performed on Control (Ctrl) and LPS‐treated cells. The experimental procedure, library construction, and high‐throughput sequencing were outsourced to and conducted by Hangzhou Guangke Ande Biotechnology Co., Ltd. (Hangzhou, China). Briefly, approximately 100,000 cells from each group were harvested and bound to Concanavalin A‐coated magnetic beads at room temperature. The bead‐bound cells were permeabilized and incubated with a specific primary antibody against H4K12la (Ptm‐biolab, PTM‐1411RM) overnight at 4°C. After stringent washing, the cells were incubated with a secondary antibody, followed by the addition of the pA‐Tn5 transposome complex. The tagmentation reaction was activated by a magnesium‐containing buffer to specifically cleave and ligate sequencing adapters at the antibody‐targeted chromatin regions. Finally, the tagmented DNA fragments were extracted, purified, and subjected to PCR amplification to generate the final sequencing libraries, which were subsequently sequenced on an Illumina platform.

### 5.3 Chromatin Immunoprecipitation (ChIP)‐qPCR Assay

To assess whether H4K12la enriches at the NDUFS7 locus, ChIP assays were performed using a ChIP kit (#91820, Cell Signaling Technology) following the manufacturer's protocols. For the ChIP assay, BV2 microglia were fixed using formaldehyde (1% final concentration) for 10 min to crosslink protein to DNA. Nuclear extracts were further isolated and subjected to enzymatic digestion and sonication to promote chromatin fragmentation. The fragmented chromatin was immunoprecipitated with anti‐H4K12la antibody (PTM‐1411RM, Ptm‐biolab) or anti‐IgG antibody (A7016, Beyotime) and then subjected to qPCR analysis. The sequences of primers for NDUFS7 are listed in Table S5.

### 5.4 Dual‐Luciferase Reporter Assay

To validate the transcriptional activity of the H4K12la‐enriched NDUFS7 peak region under LPS stimulation, the specific DNA sequence was synthesized and cloned into the pGL4.23 luciferase reporter vector. BV2 microglia were transfected with the constructed plasmids and subsequently treated with LPS. Relative luciferase activities were measured and normalized to the internal Renilla luciferase activity. The specific cloned sequence for the NDUFS7 PEAK is detailed in Table S5.

### 5.5 siRNA and Plasmid Transfection

Small interfering RNAs (siRNAs) targeting the indicated genes were synthesized by RiboBio (Guangzhou, China). BV2 microglia were transfected using Lipofectamine 3000 (Thermo Fisher Scientific, Cat. No. L3000001, USA) according to the manufacturer's protocol. RNA and protein were harvested 48 h after transfection for downstream analyses. The sequences of all siRNAs are listed in Table S2. For plasmid overexpression, mammalian expression plasmids encoding NDUFS7 and AOX were purchased from Qingke Biotechnology (Shanghai, China). HEK293T cells and BV2 microglia were transfected using Lipo8000 (Cat. No. C0533, Beyotime, China). Briefly, cells were seeded into 6‐well plates one day prior to transfection. When cell confluence reached ∼80%, the medium was replaced, and the transfection mixture was added to each well. Cells were harvested 48 h after transfection for subsequent experiments.

### 5.6 Extraction of Microglial Cell Membranes (MM)

BV2 microglial cells were harvested, resuspended in a hypotonic lysis buffer supplemented with protease inhibitors, and disrupted via ice‐bath ultrasonication. Following a differential centrifugation process (4,000 rpm for 10 min to remove intact cells and nuclei, followed by 14,800 rpm for 20 min at 4°C), the purified microglial cell membrane (MM) pellet was collected. To prepare the biomimetic MM and precisely control their size, the mixture was sequentially co‐extruded through 400 nm and 200 nm polycarbonate porous membranes (Whatman, Cytiva) for 15 cycles using a mini‐extruder, yielding the final MM with a uniform hydrodynamic diameter of approximately 140 nm.

### 5.7 Synthesis of the PEG_45_‐PPS_20_ Copolymer

The amphiphilic PEG_45_‐PPS_20_ block copolymer was synthesized via living anionic ring‐opening polymerization. Briefly, the thiol‐terminated PEG macroinitiator (mPEG‐SH, MW = 2 kDa, 1.00 g, 0.50 mmol, JenKem Technology) was dissolved in anhydrous THF (10.0 mL, Sigma‐Aldrich) under a strictly controlled nitrogen atmosphere. Sodium methoxide (32.4 mg, 0.60 mmol, Sigma‐Aldrich) was added to deprotonate the terminal thiols, generating highly reactive thiolate anions. Subsequently, purified propylene sulfide (PS) monomer (0.78 mL, 10.0 mmol, TCI Chemicals) was added dropwise into the reaction mixture. The feed molar ratio of mPEG‐SH, NaOMe, and PS was precisely maintained at 1:1.2:20 to yield a well‐defined copolymer with a targeted degree of polymerization. Upon quantitative monomer conversion, the polymerization was quenched with an excess of glacial acetic acid (0.2 mL, Sinopharm Chemical Reagent). The resulting crude product was purified by repeated precipitation in ice‐cold diethyl ether (Sinopharm Chemical Reagent) and lyophilized to obtain the final mPEG‐PPS copolymer. The chemical structure and the actual degree of polymerization of the synthesized mPEG‐PPS were verified by proton nuclear magnetic resonance (^1^H NMR) spectroscopy. The purified copolymer was dissolved in deuterated chloroform (CDCl_3_) containing tetramethylsilane (TMS) as an internal standard, and the spectra were recorded on a 400 MHz NMR spectrometer (Bruker). The actual number of PS repeating units was determined by comparing the integration area of the characteristic methyl protons (─CH_3_, δ ∼1.35 ppm) of the PPS block with the methylene protons (─CH_2_CH_2_O─, δ ∼3.64 ppm) of the PEG block.

### 5.8 Synthesis of Protac

#### 5.8.1 Step 1: Synthesis of Standard and Alkyne‐Tagged VHL Ligand‐Linker Intermediates

To generate both standard and fluorescently trackable analogues, parallel reactions were performed. A mixture of the VHL E3 ligase ligand (VH032‐amine derivative, 216 mg, 0.50 mmol, MedChemExpress), the respective linker precursor—either standard mono‐tert‐butyl dodecanedioate (172 mg, 0.60 mmol, Sigma‐Aldrich) for the standard analogue, or an alkyne‐functionalized mono‐tert‐butyl dodecanedioate (185 mg, 0.60 mmol, custom synthesized by MedChemExpress) for the tagged analogue—HATU (285 mg, 0.75 mmol, Sigma‐Aldrich), and DIPEA (261 µL, 1.50 mmol, Sigma‐Aldrich) in anhydrous DMF (5.0 mL) was stirred at room temperature overnight. The reaction mixtures were diluted with ethyl acetate, washed with brine, dried over anhydrous Na_2_SO_4_, and concentrated. The crude products were purified by silica gel chromatography to yield the Boc‐protected intermediates. These intermediates were then dissolved in DCM/TFA (4:1 v/v, 5.0 mL) and stirred for 1 h to remove the tert‐butyl protecting groups. The solvents were removed under reduced pressure to afford the parallel carboxylic acid‐terminated intermediates: VHL‐C_12_‐COOH (standard) and VHL‐C_12_(alkyne)‐COOH (alkyne‐tagged).

#### 5.8.2 Step 2: Conjugation with LDHA Warhead

A mixture of the respective intermediate—either VHL‐C_12_‐COOH (129 mg, 0.20 mmol) or VHL‐C_12_(alkyne)‐COOH (133 mg, 0.20 mmol)—the LDHA targeting warhead (CHEMBL5176448) (96 mg, 0.24 mmol, custom synthesized by MedChemExpress), HATU (114 mg, 0.30 mmol, Sigma‐Aldrich), and DIPEA (105 µL, 0.60 mmol, Sigma‐Aldrich) in DMF (3.0 mL) was stirred at room temperature for 4 h. The reactions were monitored by LC‐MS (Thermo Fisher Scientific, USA). Upon completion, the mixtures were extracted with ethyl acetate, washed with saturated NaHCO_3_ and brine, and dried. The final products were purified by preparative HPLC (Agilent Technologies, USA) to obtain the standard PROTAC and the alkyne‐tagged PROTAC, both as solid products.

#### 5.8.3 Step 3: Fluorescent Labeling via Click Chemistry for In Vivo Tracking

To enable in vivo spatial tracking, the alkyne‐tagged PROTAC (52 mg, 0.05 mmol) and Cy5.5‐Azide (38 mg, 0.06 mmol, Lumiprobe) were dissolved in a mixture of DMF/H_2_O (4:1 v/v, 2.0 mL). Catalytic amounts of CuSO_4_·5H_2_O (2.5 mg, 0.01 mmol) and sodium ascorbate (10 mg, 0.05 mmol) were added to initiate the copper‐catalyzed alkyne‐azide cycloaddition (CuAAC). The mixture was stirred at room temperature for 12 h in the dark. The reaction was monitored by LC‐MS. The resulting crude mixture was filtered and directly purified by preparative HPLC to yield the final Cy5.5‐labeled PROTAC (PROTAC‐Cy5.5).

### 5.9 IVIS Imaging

To evaluate the spatial localization and targeted delivery efficiency of the drug, PROTAC‐Cy5.5 was utilized as an intrinsic fluorescent tracer. Mice were administered equivalent doses of either free PROTAC‐Cy5.5 or PROTAC‐Cy5.5 encapsulated in mPTC and MM@mPTC at 0 Dpi. At 7 Dpi, the dorsal skin over the lesion was surgically opened under anesthesia to minimize signal scattering. Fluorescence was immediately acquired using an IVIS Spectrum system (PerkinElmer, USA). Subsequently, the animals were transcardially perfused, and major organs (spinal cord, brain, heart, liver, spleen, lungs, and kidneys) were harvested for ex vivo imaging to assess the biodistribution profiles.

### 5.10 Preparation of the mPTC Nanoparticles

The PROTAC‐loaded micelles (mPTC) were prepared using a standard nanoprecipitation and dialysis method. Briefly, 10 mg of the PEG_45_‐PPS_20_ copolymer and 1.0 mg of PROTAC molecules (or PROTAC‐Cy5.5 for the fluorescent analogues) were co‐dissolved in 1.0 mL of an organic solvent (THF, Sigma‐Aldrich). This mixture was added dropwise into 10.0 mL of deionized water under vigorous stirring to induce self‐assembly. The resulting micellar suspension was then dialyzed using a dialysis membrane tubing (MWCO: 3.5 kDa, Spectrum Laboratories) against deionized water for 48 h in the dark to completely remove the organic solvent and unencapsulated molecules. Finally, the solution was filtered through a 0.22 µm microporous membrane syringe filter (Millex, MilliporeSigma) to obtain the purified mPTC nanoparticles.

### 5.11 Preparation of the MM@mPTC Nanoparticles

To construct the biomimetic delivery system, the extracted MM pellet was resuspended in cold saline (Sinopharm Chemical Reagent, China) containing the pre‐formed mPTC micelles (or mPTC loaded with PROTAC‐Cy5.5) and briefly sonicated (Branson Ultrasonics, USA) for uniform dispersion. Subsequently, the mixture was sequentially co‐extruded through 400 nm and 200 nm polycarbonate porous membranes (Whatman, Cytiva) for 15 cycles using a mini‐extruder (Avanti Polar Lipids, USA). This mechanical extrusion process effectively camouflaged the micellar core with microglial membranes, yielding the final MM@mPTC nanoparticles with a uniform hydrodynamic diameter of approximately 140 nm.

### 5.12 Measurement of Mitochondrial NAD^+^/NADH Ratios in Cultured Cells

The NAD^+^/NADH ratio serves as an indicator of Complex I RET and was measured using an NAD^+^/NADH Assay Kit with WST‐8 (Cat. No. S0175, Beyotime, China). BV2 microglia were harvested after treatment and lysed in a buffer containing mannitol, sucrose, Tris‐HCl, and protease inhibitors. Differential centrifugation was performed to isolate mitochondria: lysates were first spun at 600 × *g* (10 min) to remove debris, and the resulting supernatant was centrifuged at 7,000 × *g* (10 min) to obtain the mitochondrial pellet. The pellet was then resuspended and extracted using the NAD^+^/NADH extraction buffer provided in the assay kit. Following the vendor's instructions, the samples were incubated with the WST‐8 working solution, and the absorbance was measured at 450 nm using a microplate reader to calculate the ratio of oxidized (NAD^+^) to reduced (NADH) nicotinamide adenine dinucleotide.

### 5.13 Assessment of Mitochondrial ROS Generation

Mitochondrial superoxide generation was assessed using MitoSOX Red (Cat. No. S0061S, Beyotime, China) and MitoTracker Green (Cat. No. C1048, Beyotime, China) according to the manufacturer's protocols. BV2 microglia and HEK293 cells were seeded in 24‐well plates (1 × 10^5^ cells/well). For genetic manipulation experiments, cells were transfected with plasmids encoding NDUFS7 or AOX (Alternative Oxidase) prior to treatment. To induce or modulate ROS production, cells were incubated with Lipopolysaccharide (LPS), Succinate (Suc), Dimethyl malonate (DMM), or FCCP at the indicated concentrations. Following treatment, cells were washed and incubated with 5 µm MitoSOX and 50 nm MitoTracker Green at 37°C for 10 min. Nuclei were counterstained with Hoechst 33342 (Cat. No. C1025, Beyotime, China). Live‐cell imaging was performed using a Nikon confocal laser scanning microscope. Fluorescence intensity was kept constant across all groups for quantitative analysis.

### 5.14 JC‐1 Detection

The mitochondrial membrane potential (ΔΨm) was evaluated using a commercial JC‐1 Staining Kit (Cat. No. M8650, Solarbio, China). Briefly, cells were washed and incubated with the JC‐1 working solution at 37°C for 20 min according to the manufacturer's instructions. After washing with the assay buffer, fluorescence images were acquired using a Nikon confocal laser scanning microscope. The ratio of red (JC‐1 aggregates) to green (JC‐1 monomers) fluorescence was analyzed to determine the changes in membrane potential.

### 5.15 Spinal Cord Injury Model

Adult mice were anesthetized with pentobarbital sodium (50 mg/kg, intraperitoneal injection), and a laminectomy was performed at T9‐T10. Spinal cord contusion was induced using a W.M. Keck impactor (5‐g weight dropped from a height of 3 cm). All animal experiments were approved by Wenzhou Medical University, China (Approval No. wydw2025‐0367). Sham‐operated mice underwent laminectomy only. Postoperative analgesia was provided with meloxicam (5 mg/kg, subcutaneous injection, once daily for three consecutive days). Postoperative care also included thermal support, manual bladder emptying twice daily, and antibiotic prophylaxis with gentamicin (5 mg/kg, subcutaneous injection, once daily for seven days). A total of 294 mice were used in this study. The animals were allocated to specific experimental groups as detailed in Table S4.

### 5.16 Behavioral Assessments

To evaluate functional recovery following spinal cord injury, behavioral tests were conducted at 28 Dpi. The Basso Mouse Scale (BMS) was utilized to assess open‐field locomotion; mice were observed for 4 min and graded on a 0–9 scale by two investigators blinded to the experimental conditions. For gait analysis, a footprint test was performed by dipping the forepaws and hindpaws in non‐toxic blue and red dyes, respectively. Stride length and toe spread were calculated based on measurements from at least three consecutive steps. Furthermore, kinematic analysis was conducted using DeepLabCut, a machine‐learning‐based tracking system, to monitor the trajectories of the hip, knee, and ankle joints. This allowed for the extraction of key parameters such as stride characteristics, swing/stance phases, and body height. Finally, to assess the conduction of descending motor tracts, motor‐evoked potentials (MEPs) were recorded. Under anesthesia, transcranial electrical stimulation was applied, and signals were captured from the gastrocnemius muscle to analyze latency and amplitude.

### 5.17 Seahorse XF Metabolic Flux Analysis

To measure the oxygen consumption rate (OCR) and extracellular acidification rate (ECAR), the Seahorse XF Cell Mito Stress Test Kit and Glycolysis Stress Test Kit were used together with the XF96 Extracellular Flux Analyzer (Agilent Technologies). BV2 microglia were seeded into Seahorse XF96 plates with glucose‐containing medium at 1 × 10^4^ cells per well and incubated overnight. For OCR measurements, oligomycin, FCCP, and rotenone/antimycin A were sequentially added according to the manufacturer's instructions. For ECAR measurements, glucose, oligomycin, and 2‐deoxyglucose (2‐DG) were added in sequence to assess glycolytic activity. OCR and ECAR data were analyzed using Wave software and plotted as mean ± SD.

### 5.18 Statistical Analysis

Data are presented as mean ± standard deviation (SD). Normality of data distribution was assessed using the Shapiro‐Wilk test. For comparisons between two independent groups, normally distributed data were analyzed using a two‐tailed unpaired Student's *t*‐test, whereas non‐normally distributed data were evaluated using the Mann‐Whitney U test. For comparisons among three or more independent groups, a one‐way analysis of variance (ANOVA) followed by Tukey's post hoc test was applied. For analyses involving two independent variables (e.g., treatment group and time point), a two‐way ANOVA with Tukey's post hoc test was utilized. The significance level was set at α = 0.05. ^*^ *p* < 0.05, ^**^ *p* < 0.01, ^***^ *p* < 0.001, and ^****^ *p* < 0.0001 were considered statistically significant. No data points were excluded as outliers. Statistical analyses were performed using R software (v4.5.0) and GraphPad Prism (v10.0). The exact sample size (n) for each experiment is indicated in the corresponding figure legend.

## Author Contributions

C.G.W., H.X., and S.R.S. designed this study. C.L.H., Z.C.J., and Z.W.W. performed the majority of the experiments and wrote the paper. C.Y.W., J.L., K.J.G., and Y.H.L. participated in experiments and revised this paper. S.B.X. helped perform animal surgery. G.L., Y.C.J., and Y.X. analyzed data and edited figures.

## Ethics Approvals

All experiments involving animals were conducted according to ethical policies, and procedures were approved by the Chinese Institutional Animal Care Committee of Wenzhou Medical University, China (Approval no. wydw2025‐0367).

## Conflicts of Interest

The authors declare no conflict of interest.

## Supporting information




**Supporting File**: advs78057‐sup‐0001‐SuppMat.docx.

## Data Availability

The data that support the findings of this study are available from the corresponding author upon reasonable request.
