# Supplementary material for: H4K12 Lactylation Regulates NDUFS7 to Drive Microglia Reverse Electron Transport in Spinal Cord Injury
Source: Adv Sci (Weinh). 2026 Sep 29:e78057. Online ahead of print. doi: 10.1002/advs.78057 (PMC13621200; doi:10.1002/advs.78057)
Supplement: Supplementary file 1 [file ADVS-9999-e78057-s001.docx]

Supplementary Materials for

**H4K12 lactylation regulates NDUFS7 to drive microglial reverse electron transport in spinal cord injury**

Chenglong Hong *et al.*

*Corresponding author: Chenggui Wang, wangchenggui@wmu.edu.cn

**This PDF file includes:**

**Supplementary Materials and Methods**

**Figure S1 to S13**

**Tables S1 to S5**

**Raw Data**

**Supplementary Materials and Methods**

**RNA Sequencing (RNA-seq) Analysis**

At 3 Dpi, spinal cord tissues encompassing the lesion epicenter were harvested from the Sham and SCI-3Dpi groups (n=3 per group). Total RNA extraction, library construction, and high-throughput sequencing (Illumina platform) were performed by Guangke Ande Biotechnology Co., Ltd. (China). Raw sequencing data were filtered to obtain high-quality clean reads, which were subsequently aligned to the mouse reference genome. Differential expression analysis was conducted using the DESeq2 package. Genes meeting the criteria of an adjusted *P*-value < 0.05 and |log2(fold change)| > 1 were defined as differentially expressed genes (DEGs).

**Proteomic Analysis**

To investigate the protein profile changes during microglial polarization, BV2 cells were treated with PBS or LPS (1ug/mL) for 24 h (n=3/per group). Cell lysis, peptide preparation, and quantitative LC-MS/MS sequencing were performed by Guangke Ande Biotechnology Co., Ltd. (China). Raw data were searched against the UniProt mouse database with a false discovery rate (FDR) < 1%. Proteins meeting the criteria of fold change > 1.2 and *P* < 0.05 were defined as differentially expressed proteins (DEPs).

**Isolation and Culture of Primary Microglia from Adult Mouse Spinal Cord**

Primary microglia were isolated from adult mouse spinal cords. Briefly, mice were euthanized and transcardially perfused with PBS. The spinal cord was extruded using an 18‑gauge needle with cold DPBS, and meninges were carefully removed under a microscope. The tissue was minced into 2–3 mm pieces and digested in an enzyme mix containing collagenase and DNase I at 37 °C for 30 min, with gentle swirling every 10 min. After adding 350 μL DNase I, the tissue was triturated sequentially with a P1000 pipette and a glass Pasteur pipette. The supernatant was collected through a 30 μm strainer, and the remaining tissue was re‑triturated and filtered. The combined suspension was centrifuged at 300 × g for 10 min. Myelin debris was removed using the debris removal solution from the adult brain dissociation kit (Miltenyi Biotec, Cat. No. 130-107-677) according to the manufacturer's instructions. Cells were resuspended in DMEM with 10% FBS and 1% penicillin/streptomycin, plated onto poly‑D‑lysine‑coated dishes (5 μg/mL), and cultured at 37 °C with 5% CO₂. After 7-10 days of culture, microglia growing on the astrocyte monolayer were detached by firmly tapping the side walls of the culture flasks with the palm for 2–5 min. The supernatant containing the detached microglia was collected, centrifuged at 300 × g for 5 min, and resuspended for downstream applications.

**Cell Culture**

BV2 microglia, HEK293T cells, and PC12 cells were purchased from Procell Life Science & Technology Co., Ltd. (Wuhan, China). BV2 microglia and HEK293T cells were cultured in high-glucose DMEM (Gibco, Cat. No. 11965092, Australia) supplemented with 10% fetal bovine serum (FBS; Gibco, Cat. No. 10099141C, Australia) and 1% penicillin-streptomycin (Gibco, Cat. No. 15140122, Australia). PC12 cells were maintained in RPMI-1640 (Gibco, Cat. No. 11875093, Australia) supplemented with 10% FBS, and 1% penicillin-streptomycin. Primary microglia isolated from adult mouse spinal cords were cultured in DMEM/F‑12 (Gibco, Cat. No. 11320033, USA) supplemented with 10% FBS and 1% penicillin‑streptomycin. All cells were maintained at 37 °C in a humidified incubator with 5% CO₂. To induce microglial activation, BV2 microglia were treated with 1 μg/mL lipopolysaccharide (LPS; MCE, HY-D1056) for 24 h.

**Cell Viability Assay (CCK-8)**

Cell viability was determined using the CCK-8 assay (Dojindo, Japan). BV2 microglia (5,000 cells/well) were plated in 96-well plates and subjected to experimental treatments. Subsequently, cells were incubated with 10 µL CCK-8 reagent for 2 h at 37 °C. Absorbance was quantified at 450 nm using a microplate spectrophotometer.

**Transwell Co-culture Assay**

A non-contact co-culture system was established using 0.4-µm Transwell inserts (Corning, USA). PC12 cells in the lower compartment were grown to confluence and scratched with a 200-µL pipette tip. After washing with PBS, BV2 microglia receiving different treatments were added to the upper inserts. After 24 h of co-incubation, the migration of PC12 cells was assessed by staining with 0.1% crystal violet (Solarbio, China) and subsequent imaging.

**Immunofluorescence Staining of Spinal Cord Tissues**

For tissue analysis, mice were transcardially perfused with PBS followed by 4% paraformaldehyde. Spinal cords were carefully dissected, post-fixed overnight in 4% paraformaldehyde at 4 °C, and then dehydrated through a graded ethanol series before being embedded in paraffin. Longitudinal sections (5 μm thickness) were cut with a rotary microtome, spanning from 2 mm rostral to 2 mm caudal of the lesion epicenter (T9-T10 level). Prior to immunofluorescence staining, paraffin sections were deparaffinized in xylene and rehydrated through a descending graded ethanol series. For antigen retrieval, sections were boiled in 10 mM sodium citrate buffer (pH 6.0) in a microwave oven for 15 min. After cooling to room temperature, sections were blocked with BlockAid™ Blocking Solution (Invitrogen, Cat. No. B10710) for 1 h at room temperature to minimize non-specific background staining.

Following blocking, sections were incubated with specific primary antibodies (Table S1) overnight at 4 °C. After washing with PBS, sections were incubated with Alexa Fluor-conjugated secondary antibodies (Table S1) for 1 h at room temperature in the dark. Nuclei were counterstained with DAPI (Yeasen, Cat. No. 36308ES20, Shanghai, China). Finally, sections were mounted with antifade mounting medium and stored at 4 °C prior to imaging.

Images were captured using a Nikon C2si confocal laser scanning microscope. For each animal, three to five sections from the lesion epicenter region (spanning 2 mm rostral to 2 mm caudal) were randomly selected for imaging. In each section, three to five fields of view (20× or 40× magnification) were acquired from the gray matter surrounding the lesion core. All images were acquired under identical laser power, gain, and exposure settings for each fluorophore across experimental groups. Quantification was performed in a blinded manner by two independent investigators. For colocalization analysis, fluorescence intensity profiles were extracted using ImageJ software (NIH), and Pearson's correlation coefficients (PCC) were calculated using the Coloc2 plugin to quantify the spatial overlap. For each marker, the mean fluorescence intensity (MFI) was measured in at least 3-5 randomly selected regions of interest (ROIs) per section, and the values were averaged per animal. A minimum of 4 mice per group (n ≥ 4) was analyzed for all immunofluorescence quantifications.

**Immunofluorescence Staining of Cells**

Immunofluorescence staining was performed on BV2 microglia seeded on coverslips. Cells were fixed with 4% paraformaldehyde (PFA) (Solarbio, Cat. No. P1110, Beijing, China) for 15 min at room temperature, permeabilized with 0.3% Triton X-100 for 20 min, and blocked with 5% bovine serum albumin (BSA) for 1 h at room temperature. Subsequently, the cells were incubated with specific primary antibodies (detailed in Supplementary Table 1) overnight at 4 °C. After washing with PBS, cells were incubated with Alexa Fluor-conjugated secondary antibodies (Supplementary Table 1) for 1 h at room temperature in the dark. Nuclei were counterstained with DAPI (Yeasen, Cat. No. 36308ES20, Shanghai, China). Finally, coverslips were mounted onto glass slides with antifade mounting medium and stored at 4 °C prior to imaging. Images were captured using a Nikon C2si confocal laser scanning microscope. For colocalization analysis, fluorescence intensity profiles were extracted using ImageJ software (NIH), and Pearson's correlation coefficients (PCC) were calculated using the Coloc2 plugin to quantify the spatial overlap.

**Dihydroethidium Staining**

The fresh frozen spinal cord sections were initially incubated with the fluorescent dye DHE (Beyotime, S0063) for ROS tracking. For further immunofluorescence co-labeling, the sections were fixed, blocked, and incubated overnight with primary antibodies. Following incubation with species-matched fluorescent secondary antibodies and DAPI counterstaining, Images were captured using a Nikon C2si confocal laser scanning microscope.

**RNA Extraction and Quantitative Real-Time PCR**
Total RNA was isolated from cells using TRIzol reagent following the manufacturer’s specifications. Complementary DNA synthesis was performed with HiScript III RT SuperMix for qPCR (Vazyme, R323-01). Quantitative real-time PCR amplification employed ChamQ Universal SYBR qPCR Master Mix (Vazyme, Q711-03) on a CFX96 Real-Time PCR system (Bio-Rad). Primer sequences are detailed in Table S3.

**AAV Delivery *in vivo***

An AAV9 vector expressing LDHA-targeting shRNA under the control of the microglia-enriched Iba1 promoter (AAV9-shLDHA, 1.82 × 10¹³ vg/mL) and the corresponding control vector (AAV9-Ctrl, 2.42 × 10¹³ vg/mL) were provided by Suzhou GeneAdv Co. Ltd. Two weeks before SCI induction, mice received intraspinal injection of AAV9-shLDHA or AAV9-Ctrl. After allowing sufficient time for viral expression, SCI surgery was performed.

**Western Blot**
 Total protein was extracted from BV2 microglia or spinal cord tissues using RIPA lysis buffer (Beyotime, No. P0013B, China) supplemented with PMSF (Beyotime, No. ST506) and a phosphatase inhibitor cocktail (Beyotime, No. P1081). After lysis on ice for 30 min, the homogenates were clarified by centrifugation at 12,000 rpm for 30 min at 4 °C. Protein concentration was quantified using the BCA Protein Assay Kit (Thermo Fisher Scientific, No. A65453, USA). Equal amounts of protein were separated by 10% or 12.5% SDS-PAGE and electrotransferred onto PVDF membranes. The membranes were blocked with 5% non-fat milk for 1 h at room temperature and then incubated with the specific primary antibodies (Table S1.) overnight at 4 °C. Subsequently, the membranes were incubated with HRP-conjugated secondary antibodies (Beyotime, Nos. A0208/A0216) for 1 h. Protein bands were imaged with a Bio-Rad chemiluminescence system.

**AAV-PHP.eB-Syn1-EGFP Injection**

To evaluate axonal regeneration following SCI, mice were intravenously injected with AAV-PHP.eB-Syn1-EGFP (1 × 10¹¹ vg/mouse in 100 μL sterile PBS; provided by Obio Technology, Shanghai, China) via the lateral tail vein immediately after injury (0 days post-injury, dpi). The PHP.eB capsid confers enhanced blood-brain barrier penetration and neuronal transduction, while the Syn1 promoter ensures neuron‑specific EGFP expression. At 28 dpi, mice were transcardially perfused, and spinal cords were harvested. Longitudinal sections were prepared and examined under a fluorescence microscope to detect EGFP‑positive axons and assess regeneration beyond the lesion site.

**Enzyme-Linked Immunosorbent Assay (ELISA)**
To evaluate the inflammatory status, the secretion levels of TNF-α, TGF-β, IL-1β, and IL-10 were assessed. Cell culture media were collected, centrifuged at 10,000 × *g* for 10 min at 4 °C, and analyze using commercial ELISA kits (Elabscience, China). The assay was performed strictly according to the manufacturer's protocol. Briefly, samples were incubated in pre-coated wells, followed by the addition of detection reagents and chromogenic substrate. Absorbance at 450 nm was recorded, and the absolute concentration of each cytokine was calculated using serial dilutions of the respective standards.

**Histological Staining**
At 28 Dpi, mice were deeply anesthetized and transcardially perfused with PBS followed by 4% paraformaldehyde (PFA) (Solarbio, Cat. No. P1110, Beijing, China). A 10-mm spinal cord segment centered on the lesion epicenter was harvested, post-fixed in 4% PFA for 24 h, dehydrated, and embedded in paraffin. Longitudinal sections (5μm thick) were prepared for subsequent staining. To evaluate general tissue morphology and cavity formation, Hematoxylin and Eosin (H&E) staining (Solarbio, Cat. No. G1120) was performed according to standard protocols. Masson’s trichrome staining (Solarbio, Cat. No. G1340) was conducted to assess fibrotic scar formation and collagen deposition. Additionally, Nissl staining (1% cresyl violet; Solarbio, Cat. No. G1430) was applied to quantify the survival of motor neurons. Whole-slide images were acquired using an Olympus VS200 digital slide scanner (Olympus, Japan).

**Electrophysiological Assessment**

To evaluate the functional integrity of descending motor pathways following spinal cord injury, motor evoked potentials (MEPs) were recorded at 28 Dpi. Briefly, mice were anesthetized with 1% pentobarbital sodium and placed on a heating pad to maintain body temperature. For transcranial stimulation, the stimulating electrode was placed subcutaneously over the motor cortex, and the reference electrode was inserted into the snout. To record the MEP signals, the recording electrode was inserted into the belly of the tibialis anterior muscle of the hindlimb, with the reference electrode positioned near the Achilles tendon. A ground electrode was placed subcutaneously at the base of the tail. Single square-wave electrical pulses (0.2 ms duration, 2.0 mA intensity) were delivered using a BL-420S biological data acquisition system. The onset latency (time from stimulus to the first deflection) and peak-to-peak amplitude of the MEPs were recorded and averaged across multiple sweeps to quantify electrophysiological recovery.

**Figure S1. Validation of the SCI model and characterization of microglial metabolic states.**

(A–B) ScRNA-seq cell clusters in the uninjured group and the SCI group at 3 days post-injury (3 Dpi). (C) Representative Western blot bands and quantification of microglia polarization markers (CD86, iNOS, CD206, and Arg-1) in spinal cord tissues from Sham and SCI groups (n=4). *p < 0.05, **p < 0.01, ***p < 0.001, and ****p < 0.0001 were considered statistically significant. (D-F) Volcano plots of differential expression from Sham vs SCI RNA-seq, Resting microglia vs pro-inflammatory microglia scRNA-seq, and Resting microglia vs pro-inflammatory microglia proteomics datasets. (G-H) Enrichment analysis of KEGG pathways and GO biological processes. Data are presented as mean ± SD. Comparisons were performed using two-way ANOVA with Tukey’s post hoc test as appropriate. *p < 0.05, **p < 0.01, ***p < 0.001, and ****p < 0.0001 vs. indicated group.

**Figure S2.** **Upregulation of CD86 and LDHA in the injured spinal cord after SCI.** (A-B) Quantification of CD86, LDHA fluorescence intensity in Sham and SCI spinal cord (n=4). (C) qPCR analysis of LDHA mRNA levels in BV2 microglia (n=3). Data are presented as mean ± SD. Comparisons were performed using unpaired t-test (two groups) as appropriate. *p < 0.05, **p < 0.01, ***p < 0.001, and ****p < 0.0001 vs. indicated group.

**Figure S3. H4K12la is specifically upregulated in microglia after SCI.** (A) Western blot analysis of eight histone lactylation sites (H2B16, H3K9, H3K14, H3K18, H4K5, H4K8, H4K12, and H4K16) in primary microglia (n=4). (B) Quantification of the relative activation level of each

histone lactylation site (n=4). (C-E) Representative immunofluorescence images of H4K12la (green) co-stained with IBA-1 (microglia marker, red), GFAP (astrocyte marker, red), or NeuN (neuronal marker, red) in spinal cord sections from Sham and SCI mice (n≥4). (F-H) Quantification of H4K12la fluorescence intensity in IBA-1⁺ microglia, GFAP⁺ astrocytes, and NeuN⁺ neurons. Data are presented as mean ± SD. Comparisons were performed using unpaired t-test (two groups), or two-way ANOVA with Tukey’s post hoc test as appropriate. *p < 0.05, **p < 0.01, ***p < 0.001, and ****p < 0.0001 vs. indicated group.

**Figure S4.** **Lactate metabolism regulates NDUFS7 expression and histone lactylation.** (A) qPCR analysis of NDUFS7 mRNA levels in BV2 microglia (n=3). (B) Representative Western blot bands of Pan-Kla, H4K12la, and NDUFS7 in BV2 microglia treated with increasing concentrations of lactate (0-20 mM) (n=3). (C-E) Quantitative analysis showing a dose-dependent increase in H4K12la, Pan-Kla, and NDUFS7 protein levels upon lactate treatment (n=3). (F) Representative Western blot bands of Pan-Kla, H4K12la, and NDUFS7 in BV2 microglia treated with increasing concentrations of 2-DG (0-20 mM) (n=3). (G-I) Quantitative analysis showing a dose-dependent decrease in H4K12la, Pan-Kla, and NDUFS7 protein levels upon 2-DG treatment (n=3). Data are presented as mean ± SD. Comparisons were performed using unpaired t-test (two groups), one-way ANOVA with Tukey’s post hoc test as appropriate. *p < 0.05, **p < 0.01, ***p < 0.001, and ****p < 0.0001 vs. indicated group.

**Figure S5. LDHA knockdown in vivo reduces H4K12la and NDUFS7 expression.** (A) Schematic illustration of the experimental timeline. (B) Representative Western blot analysis of Pan-Kla (17 kDa) in spinal cord tissues (n≥4). (C) Representative Western blot analysis of LDHA, H4K12la, and NDUFS7 expression in spinal cord tissues (n≥4). (D-F) Quantification of LDHA, H4K12la, and NDUFS7 protein expression (n≥4). (G) Quantification of relative lactate levels in spinal cord tissues (n≥4). Data are presented as mean ± SD. Comparisons were performed using unpaired t-test (two groups) as appropriate. *p < 0.05, **p < 0.01, ***p < 0.001, and ****p < 0.0001 vs. indicated group.

**Figure S6. Divergent mitochondrial membrane potential dynamics despite common superoxide accumulation in LPS-stimulated and NDUFS7-silenced in BV2 microglia.** (A) Quantification of mitochondrial superoxide levels indicated by MitoSOX Red fluorescence intensity in Ctrl, LPS, and si-NDUFS7 groups (n≥4). (B) Representative fluorescence images of JC-1 staining in Ctrl, LPS, siNDUFS7 and FCCP groups (n=3). (C) Quantitative analysis of the JC-1 red/green fluorescence ratio (n=3). (D) Time-lapse quantification of TMRM fluorescence intensity over 24 hours (n=3). Data are presented as mean ± SD. Comparisons were performed using one-way ANOVA with Tukey’s post hoc test as appropriate. *p < 0.05, **p < 0.01, ***p < 0.001, and ****p < 0.0001 vs. indicated group.

 **Figure S7.** **High mitochondrial membrane potential is a prerequisite for NDUFS7-mediated mtROS accumulation, supporting the RET mechanism.** (A) Assessment of mitochondrial superoxide levels using MitoSOX (n≥4). (B) Quantitative analysis of MitoSOX fluorescence intensity (n≥4). (C) Quantification of the JC-1 red/green fluorescence ratio (n=3). (D) Representative flow cytometry plots of JC-1 staining showing mitochondrial membrane potential (n=3). Data are presented as mean ± SD. Comparisons were performed using one-way ANOVA with Tukey’s post hoc test as appropriate. *p < 0.05, **p < 0.01, ***p < 0.001, and ****p < 0.0001 vs. indicated group.

**Figure S8.** **Succinate-driven RET dictates microglia polarization phenotypes.** (A-B) Quantitative analysis of the Western blot data shown in the main text. The expression levels of pro-inflammatory markers (iNOS, CD86) and anti-inflammatory markers (CD206, Arg-1) were measured in BV2 microglia treated with Succinate (Suc), Dimethyl malonate (DMM), or LPS alone or in combination (n=3). (C-F) qPCR analysis of mRNA expression levels for pro-inflammatory markers iNOS and COX-2, and anti-inflammatory markers CD206 and Arg-1(n=3). Data are presented as mean ± SD. Comparisons were performed using one-way ANOVA or two-way ANOVA with Tukey’s post hoc test as appropriate. *p < 0.05, **p < 0.01, ***p < 0.001, and ****p < 0.0001 vs. indicated group.

**Figure S9. LDHA knockdown blocks RET in primary microglia.** (A-B) Representative immunofluorescence images of CD86 (green), DHE (purple), DAPI (blue) and IBA-1 (red) in spinal cord (n≥4). (C-D) Quantification of CD86 and DHE fluorescence intensity in IBA-1⁺ microglia (n≥4). (E) Representative images of MitoSOX staining (red) in primary microglia isolated from AAV-Ctrl or AAV-shLDHA-treated SCI mice (n≥4). (F) Quantification of MitoSOX fluorescence intensity (n≥4). (G-I) Quantification of NAD⁺/NADH ratio, relative ATP levels, and relative lactate levels in primary microglia (n≥4). Data are presented as mean ± SD. Comparisons were performed using unpaired t-test (two groups) as appropriate. *p < 0.05, **p < 0.01, ***p < 0.001, and ****p < 0.0001 vs. indicated group.

**Figure S10. Virtual screening of the LDHA warhead and functional validation of PROTAC.** (A) Schematic flowchart illustrating the virtual screening pipeline used to identify high-affinity LDHA ligands. (B) Scatter plot distribution of LDHA ligands retrieved from the ChEMBL database. (C) Molecular docking simulations of the top 16 candidate ligands (IC50 < 10 nM) binding to the LDHA active site. (D) Detailed binding modes of the top 3 candidates with the highest RDKit scores. (E) Western blot analysis demonstrating the dose-dependent degradation of LDHA protein by the synthesized PROTAC at indicated concentrations (n=3). (F) H1-NMR spectrum of the synthesized ROS-responsive amphiphilic copolymer (PEG_45_-PPS_20_). (G) Representative images of the Transwell migration assay in PC12 cells treated with LPS + MM, LPS + mPTC, LPS + MM@mPTC (n=3). Data are presented as mean ± SD. Comparisons were performed using one-way ANOVA with Tukey’s post hoc test as appropriate. *p < 0.05, **p < 0.01, ***p < 0.001, and ****p < 0.0001 vs. indicated group.

**Figure S11.** **MM@mPTC shows excellent biocompatibility and promotes tissue preservation and functional recovery after SCI.** (A) H&E staining of major organs (Heart, Liver, Spleen, Lung, Kidney) showing no pathological toxicity, indicating the biosafety of the delivery system. (n=4). (B) Representative immunofluorescence images of the spinal cord lesion epicenter stained for GFAP (red, astrocytes/glial scar) and NF-200 (green, neurofilaments/axons) (n=4). (C-D) Quantification of immunofluorescence intensity in spinal cord sections from PBS, PTC, mPTC, and MM@mPTC groups. Tissues were stained for GFAP, and NF200 (n=4). (E) Histological assessment of spinal cord repair using H&E (lesion volume), Masson’s trichrome (fibrosis/collagen deposition), and Nissl staining (neuronal survival) (n=4). Data are presented as mean ± SD. Comparisons were performed using one-way ANOVA with Tukey’s post hoc test as appropriate. *p < 0.05, **p < 0.01, ***p < 0.001, and ****p < 0.0001 vs. indicated group.

**Figure S12. MM@mPTC blocks RET in primary microglia.** (A) Representative images of MitoSOX staining (red) in primary microglia isolated from PBS, PTC, mPTC, MM@mPTC-treated SCI mice (n=4). (B) Quantification of MitoSOX fluorescence intensity (n=4). (C-E) Quantification of NAD⁺/NADH ratio, relative ATP levels, and relative lactate levels in primary microglia (n=4). Data are presented as mean ± SD. Comparisons were performed using one-way ANOVA with Tukey’s post hoc test as appropriate. *p < 0.05, **p < 0.01, ***p < 0.001, and ****p < 0.0001 vs. indicated group.

**Figure S13.** **MM@mPTC preserves neurons in the peri-lesion area after SCI.** (A) Representative images of NeuN (red) staining used to assess neuronal survival. To precisely quantify neuron loss, spinal cord sections were subdivided into four distinct zones (Z1-Z4) based on their distance from the lesion center (n=4).

**Supplementary Tables**

Table S1.

Antibodies information.

| Antibodies | Company | Catalog# | Application/Dilution |
| --- | --- | --- | --- |
| LDHA | Abclone | A0861 | WB (1:1000); IF (1:200); |
| NDUFS7 | Abclone | A24466 | WB (1:1000); IF (1:200); |
| iNOS | Proteintech | 18985-1-AP | WB (1:1000) |
| CD86 | Santa Cruz | sc-19617 | WB (1:200); IF (1:200) |
| Arg1 | Proteintech | 16001-1-AP | WB (1:1000); IF (1:200) |
| CD206 | Proteintech | 18704-1-AP | WB (1:1000); IF (1:200) |
| Beta actin | Abcam | ab6276 | WB (1:10000) |
| Pan Lactic acid-Lysine | Abclone | A23004 | WB (1:1000); IF (1:200) |
| L-Lactyl-Histone - H2B (Lys16) | Ptm-biolab | PTM-1424RM | WB (1:2000); |
| L-Lactyl-Histone H3 (Lys9) | Ptm-biolab | PTM-1419RM | WB (1:2000); |
| L-Lactyl-Histone H3 (Lys14) | Ptm-biolab | PTM-1414RM | WB (1:2000); |
| L-Lactyl-Histone H3 (Lys18) | Ptm-biolab | PTM-1427RM | WB (1:2000); |
| L-Lactyl-Histone H4 (Lys5) | Ptm-biolab | PTM-1409RM | WB (1:2000); |
| L-Lactyl-Histone H4 (Lys8) | Ptm-biolab | PTM-1415RM | WB (1:2000); |
| L-Lactyl-Histone H4 (Lys12) | Ptm-biolab | PTM-1411RM | WB (1:2000); |
| L-Lactyl-Histone H4 (Lys16) | Ptm-biolab | PTM-1417RM | WB (1:2000); |
| Histone H3 | Ptm-biolab | PTM-1001RM | WB (1:2000); |
| Histone H4 | Ptm-biolab | PTM-1015RM | WB (1:2000); |
| IBA-1 | Abcam | ab318302; ab178846 | IF (1:200) |
| GFAP | Abcam | ab4648 | WB (1:2000); IF (1:200) |
| NF200 | Abcam | ab207176 | IF (1:200) |
| MBP | Proteintech | 10458-1-AP | IF (1:200) |
| NeuN | Abcam | ab177487 | IF (1:200) |
| TSG101 | Wanleibio | WL05130 | WB (1:1000) |
| CD9 | Affinity | AF5139 | WB (1:1000) |
| Alix | Wanleibio | WL03063 | WB (1:1000) |
| CD81 | Proteintech | 27855-1-AP | WB (1:1000) |
| Calnexin | Affinity | AF5362 | WB (1:1000) |
| Goat Anti-Rabbit IgG H&L (Alexa Fluor®488) | Abcam | ab150077 | IF (1:200) |
| Goat Anti-Rabbit IgG H&L (Alexa Fluor®647) | Abcam | ab150083 | IF (1:200) |
| Goat Anti-Mouse IgG H&L (Alexa Fluor® 488) | Abcam | ab150113 | IF (1:200) |
| Goat Anti-Mouse IgG H&L (Alexa Fluor® 594) | Abcam | ab150116 | IF (1:200) |
| Goat Anti-Chicken IgY H&L (Alexa Fluor® 594) | Abcam | ab150176 | IF (1:200) |
| Goat anti  Mouse IgG  (HRP  conjugated) | Beyotime | A0216 | WB (1:1000) |
| Goat anti  Rabbit IgG  (HRP  conjugated) | Beyotime | A0208 | WB (1:1000) |

Table S2.

Sequence of SiRNAs.

| SiRNAs | |
| --- | --- |
| Si-NDUFS7#1 | GCGCTATGACATGGACCGCTT |
| Si-NDUFS7#2 | GCAGAGGAAGATCAAGCGG |
| Si-LDHA#1 | GGAGAAAGCCGTCTTAATT |

Table S3.

Primers utilized for RT-qPCR

| Primers utilized for RT-qPCR | |
| --- | --- |
| CD206 | F: CTCTGTTCAGCTATTGGACGC  R: TGGCACTCCCAAACATAATTTGA |
| Arg-1 | F: CTCCAAGCCAAAGTCCTTAGAG  R: AGGAGCTGTCATTAGGGACATC |
| iNOS | F: GTTCTCAGCCCAACAATACAAGA  R: GTGGACGGGTCGATGTCAC |
| COX-2 | F: TGAGCAACTATTCCAAACCAGC  R: GCACGTAGTCTTCGATCACTATC |
| NDUFS7 | F: GTTCATCAGAGTGTAGCCACTG  R: CAGGCCGAAGGTCATAGGC |
| LDHA | F: TGTCTCCAGCAAAGACTACTGT  R: GACTGTACTTGACAATGTTGGACA |
| β-actin | F: CAAGTACTCTGTGTGGATCGGT  R: ACGCAGCTCAGTAACAGTCCG |

Table S4.

Experimental groups and sample sizes

| Summary of experimental groups and the number of mice used. | | |
| --- | --- | --- |
| Experimental Group | Experiment | Sample Size (n) |
| Sham | WB | 6 |
| Sham | IF | 13 |
| Sham | Behavioral assessments | 8 |
| Sham | MEPs | 6 |
| Sham | RNA-seq Analysis | 3 |
| Sham | Lactate assay | 6 |
| Sham | Isolate microglia | 4 |
| SCI+AAV-Ctrl | WB | 12 |
| SCI+AAV-Ctrl | IF | 6 |
| SCI+AAV-Ctrl | Lactate assay | 6 |
| SCI+AAV-Ctrl | Isolate microglia | 6 |
| SCI+AAV-shLDHA | WB | 12 |
| SCI+AAV-shLDHA | IF | 6 |
| SCI+AAV-shLDHA | Lactate assay | 6 |
| SCI+AAV-shLDHA | Isolate microglia | 6 |
| SCI+AAV-PHP.eB-Syn1 | IF | 20 |
| SCI-3Dpi | WB | 6 |
| SCI-3Dpi | IF | 13 |
| SCI-3Dpi | RNA-seq Analysis | 3 |
| SCI-3Dpi | Lactate assay | 6 |
| SCI-3Dpi | Isolate microglia | 4 |
| SCI-28Dpi (PBS) | IF/Histological staining | 8 |
| SCI-28Dpi (PBS) | IVIS Imaging | 6 |
| SCI-28Dpi (PBS) | Behavioral assessments | 8 |
| SCI-28Dpi (PBS) | MEPs | 6 |
| SCI-28Dpi (PBS) | Isolate microglia | 6 |
| SCI-28Dpi (PTC) | IF/Histological staining | 8 |
| SCI-28Dpi (PTC)  (PROTAC-Cy5.5) | IVIS Imaging | 6 |
| SCI-28Dpi (PTC) | Behavioral assessments | 8 |
| SCI-28Dpi (PTC) | MEPs | 6 |
| SCI-28Dpi (PTC) | Isolate microglia | 6 |
| SCI-28Dpi (mPTC) | IF/Histological staining | 8 |
| SCI-28Dpi (mPTC)  (PROTAC-Cy5.5) | IVIS Imaging | 6 |
| SCI-28Dpi (mPTC) | Behavioral assessments | 8 |
| SCI-28Dpi (mPTC) | MEPs | 6 |
| SCI-28Dpi (mPTC) | Isolate microglia | 6 |
| SCI-28Dpi (MM@mPTC) | IF/Histological staining | 8 |
| SCI-28Dpi (MM@mPTC)  (PROTAC-Cy5.5) | IVIS Imaging | 6 |
| SCI-28Dpi (MM@mPTC) | Behavioral assessments | 8 |
| SCI-28Dpi (MM@mPTC) | MEPs | 6 |
| SCI-28Dpi (MM@mPTC) | Isolate microglia | 6 |

Table S5.

Primers and Genomic Sequences used for ChIP-qPCR and Luciferase Reporter Assays

| Primers and Genomic Sequences | | |
| --- | --- | --- |
| Target Gene | Application | Sequence |
| NDUFS7 | ChIP-qPCR | F: TGGTCCCCAAGCTCTCTCAT  R: CGTGCCCAGTTGATGAGGT |
| NDUFS7 | Luciferase Assay | F: CGGGGTACCGTGTAGGCCTTGTGGACCTC  R: CCGCTCGAGGACTTACCCGGCGTGCCCAG |

**Uncropped WB blot**

**Original microscope images**
